# A Review on Lead-Free Hybrid Halide Perovskites as Light Absorbers for Photovoltaic Applications Based on Their Structural, Optical, and Morphological Properties

**DOI:** 10.3390/molecules25215039

**Published:** 2020-10-30

**Authors:** Shadrack J. Adjogri, Edson L. Meyer

**Affiliations:** 1 Fort Hare Institute of Technology, University of Fort Hare, Alice 5700, South Africa; emeyer@ufh.ac.za; 2 Department of Chemistry, University of Fort Hare, Alice 5700, South Africa

**Keywords:** metal halide perovskites, solar cells, morphology, bandgap, composition, photovoltaic, efficiency, absorbers, lead-free, low dimensional materials

## Abstract

Despite the advancement made by the scientific community in the evolving photovoltaic technologies, including the achievement of a 29.1% power conversion efficiency of perovskite solar cells over the past two decades, there are still numerous challenges facing the advancement of lead-based halide perovskite absorbers for perovskite photovoltaic applications. Among the numerous challenges, the major concern is centered around the toxicity of the emerging lead-based halide perovskite absorbers, thereby leading to drawbacks for their pragmatic application and commercialization. Hence, the replacement of lead in the perovskite material with non-hazardous metal has become the central focus for the actualization of hybrid perovskite technology. This review focuses on lead-free hybrid halide perovskites as light absorbers with emphasis on how their chemical compositions influence optical properties, morphological properties, and to a certain extent, the stability of these perovskite materials.

## 1. Introduction

The evolving photovoltaic technologies have recently made possible the change of sunlight into electrical energy with elevated power conversion efficiencies at little expense [1]. Electrical energy is a highly suitable and harmless energy for daily human utilization. The industrial and consumer need for electrical energy is quickly growing. Hence, the need for continuous production of electrical energy from the sun’s energy is of paramount importance since it is unpolluted, renewable, plentiful, and natural [2]. Amongst the emerging photovoltaic technologies, perovskite solar cells have speedily ascended to the frontline as possible devices for electricity production [3,4]. Perovskite solar cells hold recorded high conversion efficiency of 29.1% in the photovoltaic research field [5]and are of low cost [6,7,8,9].

Perovskite compounds are made from different inorganic and organic material compositions [10]. Hybrid perovskite materials occur primarily as metal halide coordinated semiconductors by the common formulation of (RNH_3_) MX_3_ (R = C_n_H_2n+1_; X = halogen I, Br, Cl; M = Pb, Sn, Ge, etc.) with exploratory attributes of forming varied chemical composition and structures. They have the intrinsic tendencies of forming cubic composition (space group *Pm3^−^m*), thereby being characterized through the regularly assembling solid ABX_3_, in which B represents the metal positively charged ion, and X denotes a halogen negatively charged ion [11]. During the past century, the creative family of these compounds has been duly researched, and it was discovered that they possess unique physical properties that are of great benefit to material science. The noteworthy physical properties are colossal magnetoresistance, ferroelectricity, and superconductivity [12]. By extension of their versatility, the materials are known to have unique photophysical properties which include tunable bandgap, a high absorption coefficient, long charge carrier (electron–hole) diffusion length, and low-temperature solution processability [13]. At different operating conditions, the perovskite materials are formed through synthesis, purification, and identification processes whereby at very high temperatures in the range of greater than 1300 K, they are routinely synthesized by solid-state mixing of constituent elements or compounds. Moreover, they have been synthesized through other methods such as by dehydrating the solution of precursor salts wherein the ones presenting expressions of semiconductor characteristics display significant application in the area of printable electronics due to their solution processability [14]. In the field of photovoltaics, the chemical composition of perovskite compounds is known to control the photovoltaic and optical properties of perovskite solar cells [15,16].

By stringent rules and standards, perovskite materials are being designed by using measurable confirmation tools to form unique chemical compositions using desired component properties. The measurable spatial arrangements of ABX_3_ perovskite compounds are conceivably being designed using the Goldschmidt tolerance (t) and the octahedral factors (μ). The Goldschmidt tolerance can be expressed by t = (R_A_ + R_B_)/√2(R_B_ + R_X_), in which R_A_, R_B_, and R_X_ stand for the ionic radii of A, B, and X ions, respectively [15,17,18,19,20,21,22,23,24]. The octahedral factor is shown as μ = R_B_/R_X_ [15,18]. The chemical interactions between constituent elements and ions are not accounted for by empirical schemes of the tolerance and octahedral factors. The structure of halide crystals would be empirically designed when t ranges from 0.813 to 1,107 and μ from 0.442 to 0.895.38. The assumption that the ionic radius is constant regardless of temperature and that ions are rigid spheres, including organic ions, is used for the calculation of the tolerance and octahedral factors [15]. Therefore, it is well known that a metal halide perovskite can be formed based on three peculiar factors: (1) the materials must be made up of anions and cations, in which there must exist a charge neutrality between the negatively charged ions and positively charged ions; (2) the materials’ octahedral factor must be calculated whereby the octahedral factor μ tends to envisage the stability of the BX_6_ octahedron; and (3) the material formation strategy must meet the basic features of the Goldschmidt tolerance factor t for the ionic radii of A, B and X [25].

Due to their outstanding electron–photon conversion efficiency, simplicity of assembly, and remarkable tolerance defects, a significant interest in the study of metal halogen-centered perovskite compounds has been witnessed in the past decades. In particular, the Pb-based halide perovskites in solar cells have shown high performance due to high absorption and emission efficiencies in connection with direct bandgaps [26]. Nevertheless, the significant limitations of Pb-based halide perovskites for their practical usage and marketability are due to the harmfulness of lead compounds and the low photostability of lead-based halide perovskites [27]. Over the years, intensive research has been carried out in a bid to mitigate the problem of lead in the industry. Based on the purpose of overcoming the limitations, theoretical studies have shown that the perovskite composition and the dormant Pb 6s subshells are partially accountable for the excellent photovoltaic characteristics of the Pb-based halide perovskites. Research has shown that other cations comprise dormant outward subshell s energy levels and that they are environmentally benign. These cations, Ge^2+^, Sn^2+^, Sb^3+^, Bi^3+^, and Cu^2+^, substitute Pb^2+^ to form Pb-free perovskites. As such, the growing exigency in the non-hazardous constituents of light-harvesting compounds has recently led towards the advancement of Pb-free perovskite solar cell devices [28].

The progress in perovskite coordination can be understood in two ways: morphological optimization and compositional coordination. The morphological optimization was enabled by the basic conception of the photo-electrical characteristics of perovskite. From previous research findings, it has been depicted that perovskite morphology has been changed from dots to bulk in the very recent state-of-the-art technology. In terms of compositional coordination, single cation-based and single halide-based perovskites have changed into structures with multiple cations and anions to provide superior properties and stability [29]. Furthermore, through tailoring of the chemical configuration, nanostructuring, and quantum confinement, bandgap engineering in the class of materials is achieved accordingly [30]. This research, therefore, shall focus on using chelating nitrogen materials for morphological optimization and compositional engineering to provide superior photo-electrical properties and stability.

This review article mainly discusses the replacement of lead via both homo and heterovalent replacement and their impact on the halide perovskite materials and photovoltaic properties. Furthermore, the collective outcomes from the investigational studies on the lead-free metal perovskite are discussed with emphasis on how chemical composition influences optical and morphological properties, and limitations for future studies are identified.

### 1.1. Pb Content of Perovskite and Crystalline Silicon Solar Cells

Presently, the most commonly operated and the more stable perovskite solar cell (PSC) devices utilize Pb salts as light-harvesting materials, but these lead salts contaminate the environment with an intense impact on human health. In the scientific community, the argument concerning the environmental effect of halide perovskites is still generating concerns and currently remains under debate because systematic investigations are not available [31]. Scientists have pointed out that one of the possible ways to resolve the problem of toxicity is through the reduction of the quantity of Pb salt in halide perovskite. Nevertheless, the question of how much Pb is accommodated in perovskite solar cells remains. Another vital question is: can this quantity be compared with lead-incorporating devices such as batteries? Life cycle assessment has been improvised as a functional unit to analyze environmental impacts caused by solar cell devices via the comparison of different devices and applications. For instance, a risk assessment using the “functional unit” has been used to analyze the Pb content in benchmark CH_3_NH_3_PbI_3_-based perovskite solar cells. An evident meaning of the “functional unit” is whereby calculations are performed on the quantity of electric current generated (1 kWh) by way of solar cells as well as through the disturbance that is required to undertake a lifetime on behalf of the solar cells [32].

Li and co-workers recently came up with an estimation procedure to determine the Pb content in PSCs, whereby they used three different perovskite compositions to exhibit the evaluation of Pb content. A calculation was carried out in order evaluate the lead content in the MAPbI_3_ perovskite composition by using parameters such as molar mass (620 gmol^−1^), the mass percentage of Pb (207/620 = 33.39%), and density (4.09 gcm^−3^) to give the required mass of Pb per unit area, which stands at around 0.75 gm^−2^ in support of a typical 550 nm-thick film. Likewise, FAPbI_3_ has a calculated mass of about 0.74 g m^−2^ of Pb per unit area when using a 32.7% Pb mass percentage and 4.10 g cm^−1^ density. From the weighted average of a mixture of MAPbBr_3_ (3.83 gcm^−3^), FAPbI_3_ (4.10 gcm^−3^), and CsPbI_3_ (5.39 gcm^−3^), a hybrid perovskite structure of (CsPbI_3_)_0.05_(FAPbI_3_)_0.85_(MAPbBr_3_)_0.15_ was estimated to have a 33.7% mass percentage of Pb and a 4.12 gcm^−3^ density, thereby resulting in the mass of Pb per unit area being around 0.76 gm^−2^ [33].

Meanwhile, the Pb content is measured in relation to its unit area concentration of Pb valued at around 0.75 gm^−2^ based on a standard 550 nm-thick lead (Pb)-based PSC, and it is known to be one hundred times greater in magnitude compared to that of current-day Pb content in paints valued at around 0.007 gm^−2^. Nevertheless, it is a degree of magnitude smaller than that for Pb-based paints (around 10 gm^−2^) which resulted in it being outlawed. When the perovskite devices are subjected to mild environmental damage, improved material insulation can decrease Pb outflow from such appliances. However, the development of desirable strategies can reduce or avoid the possible Pb outflow on the way to the surroundings in the case of air, groundwater, or soil. It is well known that designs aimed at glass insulation to tackle combustion hazards have been recorded, but the water opening to the water-susceptible perovskite film and the successive unalterable leakage of Pb in the direction of flow to groundwater and/or soils are focused on [33].

For the manufacture of Si solar cells, metallization exists as a key process step [34]. To date, the fabrication of nearly all the commercial Si solar cells has been achieved through the metallization of screen-printed silver (Ag) paste and at a minimum 20% boost in efficiency through the refining process of Ag paste metallization. Ag paste is prepared with three constituents: Ag powder, glass frit, and an organic carrier, whereby in the course of preparation, the glass frit has its content tuned from 0% to 2.5 weight%, and the Ag powder as well as the organic carrier are mixed at 87.5–90 weight% and 7.5–10 weight%, respectively. The organic carrier is a combination of a solvent (e.g., terpilenol), thickener (e.g., ethocel), plasticizer (e.g., phthalate), surfactant (e.g., caprylic acid), thixotropic agent (e.g., hydrogenated castor oil), and other additives [35]. The glass frit is a Pb-based compound and is a fundamental constituent in conductive silver dense film pastes that carry out an essential function in the metallization development of the pastes that have been employed widely in fusion circuits, solar energy cells, microelectronic packages, and other appliances based on outstanding electrical performance [36].

The average deduction of life cycle assessment studies, notwithstanding these deviations in lead impact effects, states that lead-based PSCs do not cause more inconveniences that obstruct substantial production and distribution when related to other marketable photovoltaic know-hows, such as crystalline silicon solar cells in the valuation of an incomplete product life cycle from resource extraction to the factory [32].

### 1.2. Composition Engineering

Composition engineering has proven to be an active approach to tailor the characteristics of perovskites and enhance the functioning of PSCs. The merits of perovskites with different cations are combined due to the mixing of the monovalent cations, which is one of the greatest commonly employed techniques in composition engineering of most perovskite materials [37]. Through the use of different incorporation of various elements, perovskites can form multi-layered compositions on the same chemical formula, which makes them versatile and highly attractive [38]. Therefore, the designs of organic–inorganic halides for specific applications, including materials for lighting and scintillators, can be achieved through the control of structural dimensionality and chemical compositions. Using the formation of mixed halide, A–B–X (A = organic positively charged ions, B = metallic positively charged ions, X = halide negatively charged ions) for the consideration of compositional and structural tunability, a variety of substitutions based on this principle could be effected out by focusing on each of the A, B and X positions. The control of the compound’s dimensionality can be achieved through the modification of organic cation magnitude through A-site replacement, thereby allowing thee materials to be liberally changed from 0 cluster combinations to 3D systems. By adding to the magnitude effects to compositional engineering of hybrid halide ABX_3_, the substitution of B and X sites permits fine-tuning of electronic configuration and optoelectronic characteristics since the B and X component states are the leading donors to the state regarding the Fermi level [39].

The perovskite arrangement of both the crystal composition and ion governs its structural, optical, and electronic properties, thereby establishing their structure–property relationship [38]. For instance, the various compositions of the perovskite-performing layer will change their respective trait and photophysical effects of the compound films. Moreover, the concentration of reactants in the solution has a superior influence on the morphology of the perovskite film, which eventually impacts the conversion efficiency of the solar cells [40].

### 1.3. Bandgap Engineering

Solar energy is intensely directed in the visible and near-infra-red (NIR) regime. Therefore, the choice of perovskite materials as a solar absorber for light-harvesting relies on the broad and intense absorption above the visible to the near infra-red domain of the solar spectrum [10,41]. The bandgap is the basic characteristic of a light harvester which controls the maximum theoretical power conversion efficiency. It is recognized to possess direct control by way of the actual performance of the perovskite cell device. It is also a property that determines whether the perovskite compound can either absorb the light particle within the visible spectrum or decline the absorption [42]. Since the bandgap is directly accountable for determining the potency (voltage) of the solar cell electric field, coupled with a situation in which the bandgap is too small, the device is able to collect extra current at the expense of possessing a small open-circuit voltage (V_OC_). However, in a situation in which the bandgap is too wide, such as 2 eV, it is a minor portion of the solar energy that can be absorbed. Therefore, an ideal bandgap tailored for a semiconductor stands at about 1.4–1.6 eV, and the same optical value is fined tuned for solar cells which are usually exhibited by distinct compounds [10,41].

The tunability of a direct bandgap is directly controlled by the composition choices of metal, halogens, and organic cations. Meanwhile, the compositional choices of metal, halogens, and organic cations can also lead to a variety of perovskites with different properties [43]. There are two major strategies for bandgap tuning: (i) Through chemical modification that entails changing the halogens (I−, Br−, Cl−). Through the atomic number progression of halogens, the electronegativity is reduced, thereby becoming similar to the values of the transitions metals with s^2^ electronic configuration, excluding lead (II) due to toxicity. (ii) Through chemical modification that entails the changing of the organic cation which is known for tuning the bandgap. Depending on their size, the organic cation controls the degree of metal halide orbital overlap, thereby causing the A-site to alter the valance and conduction band energies as well as the bandgap [44]. Due to the changing of organic cations, the other properties of the hybrid perovskite are manipulated, such as stability, charge separation abilities, carrier transport, etc. [45]. All of the perovskite lattice sites can undergo chemical substitution, therefore, it is essential to identify the chemical peculiarity amidst the three methods. For instance, the 3D perovskite with a common formula of ABX_3_ shows the importance of rich chemical replacement. A-site substitution of the perovskite material does not precisely provide the frontline electronic configuration, but it can exhibit an implied control by varying the crystal composition. Meanwhile, replacement at the B-site plays a major role in altering the conduction band, which, in turn, alters the electronic characteristics of the material. For X-site substitution, the anions dictate the valence band energy. Upon halide substitution of the perovskite material, the observed bandgap changes due to the influence through the energy levels of the negatively charged ions that are moving by way of the Cl–Br–I hierarchical pattern, whereby the energy bandgap changes due to the variation in the valence band structure that is moving by way of the 3p–4p–5p hierarchical orbital pattern with a monotonic reduction in the minimum amount of energy required to remove the electrons from the chemical atoms as the negatively charged electrons [46].

### 1.4. Morphology Engineering

Morphology, according to IUPAC, signifies shape, optical appearance, or form of phase domains in materials. From nanoscale to the macroscale, morphology is well known to perform a fundamental responsibility in the performance and properties in regard to the field of halide perovskites [47]. Therefore, the overall photovoltaic device performance significantly centers on the morphology, stoichiometry, and crystallinity of the materials [48]. In other words, perovskite layers must have distinct granule configuration, broad surface coverage, and small surface roughness to allow for recognition of an efficient solar cell [49]. As such, the deposition method of the perovskite material is of paramount importance, as confirmed by the experimental works of Burschka et al. and Bi et al. showcasing all the treatments and conditions that the materials undergo as part of and/or following the conversion to the final perovskite form. For instance, a recent report on power conversion efficiencies (PCEs) of over 15% from a planar model heterojunction perovskite solar cell was attributed to the development of extremely homogeneous flat films of the hybrid halide perovskite by vapor deposition [48].

The preparation of the perovskite thin films can be performed by various procedures such as vacuum evaporation deposition, solution-based methods, and the low-temperature vapor-assisted solution process. The dual-stage solution route deposition procedure was demonstrated to be the best amidst the various techniques, particularly due to its usefulness for preparing films of organic−inorganic complexes, in which the organic part is hard to evaporate, and/or for complexes in which the inorganic and organic parts have unsuitable solubility properties [49]. Furthermore, Sanders et al. reported that to optimize the morphology of perovskite compounds, the control of the perovskite precursors’ concentration in solution and that of the rotation speed should be particularly implemented [50].

## 2. Single Perovskite Absorbers (ABX_3_)

**Figure 1:**
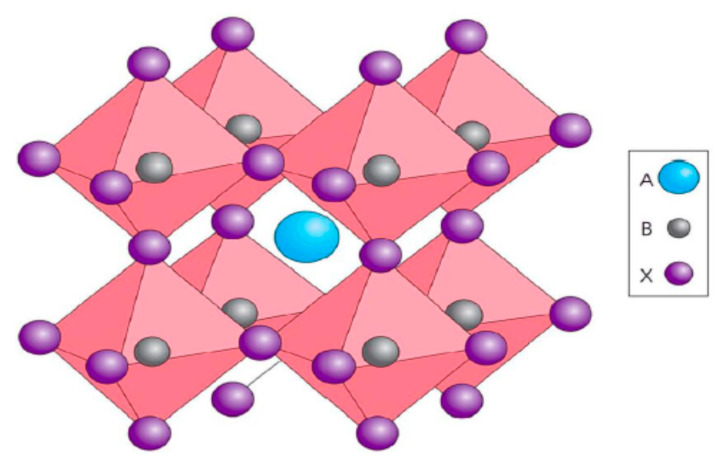
A cubic crystal perovskite composition wherein the large A cation is a monovalent, non-bonding positively charged ion, such as Cs, CH_3_NH_3_ or HC(NH_2_)_2_ [51]; B is a bivalent metallic ion (mainly Pb^2+^, Sn^2+^, Eu^2+^, Cu^2+^, Ge^2+^, etc.); and X is a halogen negatively charged ion bonded to the metal (including (F−, Cl− Br−, and I− [52].) Reproduced with permission from [53]. Copyright: Nature Photonics, Macmillan Publishers Limited (2014).

The single perovskites are derived from the common formula of ABX_3_, whereby a monovalent is identified as A in the place of non-bonding positively charged ions, such as Cs, CH_3_NH_3_ or HC(NH_2_)_2_; a bivalent metal positively charged ion remains depicted as B for mainly Pb^2+^, Sn^2+^, Eu^2+^, Cu^2+^, Ge^2+^, etc.; and X represents a halogen negatively charged ion bonded to the strategically located metal, which is seen to include (F−, Cl− Br−, and I−). A perfect simple cubic crystal lattice is constructed by anionic 3D networks of corner-sharing (MX_6/2_) octahedra organo-metal halide perovskites, as shown in Figure 1. The self-assembly of the inorganic units is occupied by the monovalent cations which generate cuboctahedral cavities, including balancing the charge and indirectly dictating the long-range structural properties. To date, the A-sites with positively charged ions, including Cs, CH_3_NH_3_, or HC(NH_2_)_2_, are only capable of stabilizing 3D structures [51,52]. The narrow selection of A cations can establish the stabilization of 3D structure since the space of the inorganic composition can only keep hold of sizeable cations that are derived from the determinant tolerance factor [51]. From a structural standpoint, Sn and Ge are the only bivalent metal cations that can substitute for the toxic Pb^2+^ in single perovskites to form a three dimensional (3D) perovskite framework with properties of uniform values along all axes in, addition to displaying equivalent charge carrier transport features equally observed in the case of lead-based perovskites [27]. Therefore, Sn−and Ge−based perovskites are the two single perovskites that form a three dimensional (3D) perovskite framework. Herein, a summary of device performance on selected single perovskite light absorbers with general formula ABX_3_ based on 3D lead-free hybrid perovskite solar cells is displayed in Table 1.

### 2.1. D ABX_3_ Metal Halide Perovskites and Perovskite-Related Absorbers with Diverse Dimensionalities

**Figure 2:**
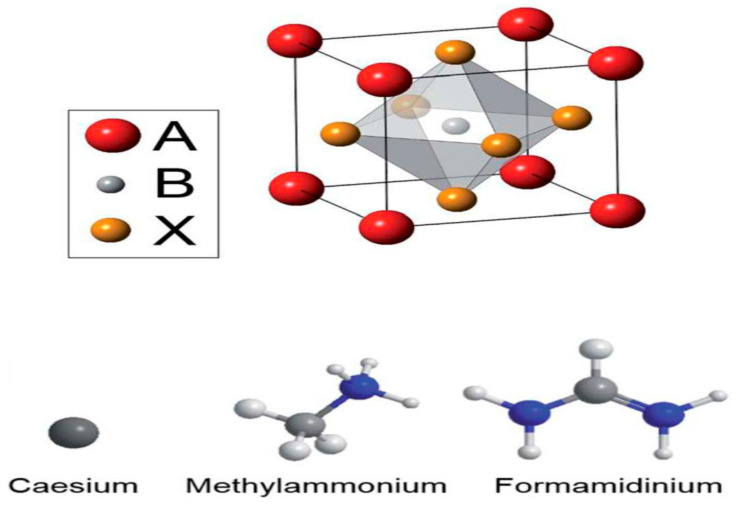
The crystal structures of the 3D ABX_3_ organometal halide perovskites in the pristine phases and the atomic composition of the three A positively charged ions studied. Reproduced with permission [68]. Copyright: The Royal Society of Chemistry (2014).

The crystallographic form of the 3D ABX_3_ organometal halide perovskites consists of corner-sharing BX_6_ octahedra with the A component neutralizing the overall charge, whereby the A component stands for organic positively charged ions, B represents the metal, and X halide negatively charged ions. The crystal structures of the 3D ABX_3_ organometal halide perovskites in the pristine phases and the atomic composition of the three A positively charged ions studied are as shown in Figure 2 [68].

**Figure 3:**
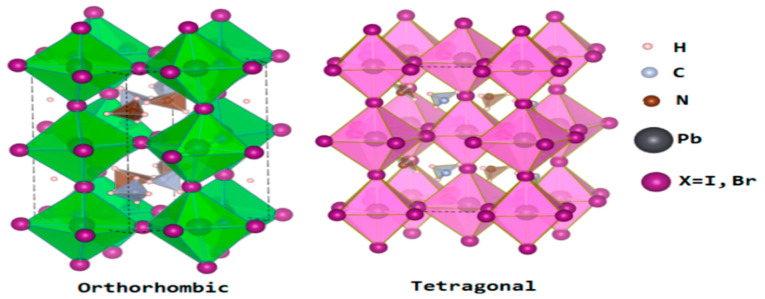
The crystal structures of the 3D ABX_3_ organometal halide perovskites in the orthorhombic and tetragonal phases. Reproduced with permission [70]. Copyright: American Chemical Society (2014).

At lower temperatures, the distortion of the BX_6_ having a B–X–B bond angle of 180 as well as ions in the interstices in regard to the 3D organometal halide perovskite lattice may be in orthorhombic phases. Hence, a function of temperature was found to cause the transition from orthorhombic to tetragonal to cubic perovskite structures. As a major function of temperature, the crystal structures of the 3D ABX_3_ organometal halide perovskites usually transit into the orthorhombic and tetragonal phases, as shown in Figure 3 [43]. These phase transitions can be influenced by various parameters, such as the precise stoichiometry of the perovskite or external limitations [69].

**Figure 4:**
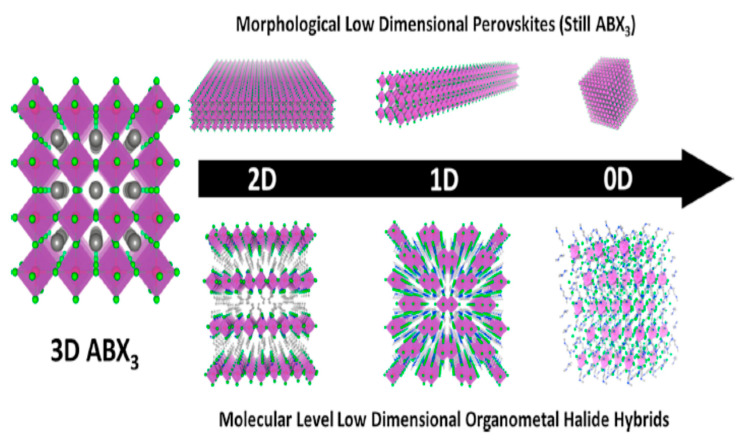
3D ABX_3_ halide perovskites and perovskite-related absorbers with diverse dimensionalities at both morphological and molecular levels. Reproduced with permission [71]. Copyright: Elsevier Ltd. (2018).

In the crystallographic form of the 3D ABX_3_ organometal halide perovskites, the A cation does not determine the band structure, but its magnitude is very crucial, whereby A being bigger or smaller could affect the expansion or contraction of the whole lattice. Moreover, the A cation appears to occupy charge neutrality within the lattice. Meanwhile, changing the B–X bond length is solely responsible for determining the bandgap. Based on the fact that a cation should appropriate amid the corner-sharing metal halide octahedra, a specific metal and halide must be known based on the fact that there exists a reasonably lesser magnitude range permitted for the A positively charged ions. Supposing A positively charged ions are extremely outsized, the 3D perovskite crystal becomes unsuitable, thereby leading to the formation of lower-dimensional layered perovskites, as shown in the Figure 4. On the other hand, in a situation where the A cation is too small, the lattice would be overly constrained to take shape [68].

### 2.2. Sn-Based 3D Perovskite Absorbers (ASnX_3_)

Sn belongs to the carbon family of group IV in the periodic table of elements, and as a monoatomic ion, it has a radius of 110 pm. It is associated with a member of Pb (119 pm), which is composed to formulate the ASnX_3_ perovskite compound, where A stands for MA^+^, FA^+^, and Cs^+^ positively charged ions and X is a halogen negatively charged ions in analogy to APbX_3_ perovskite [72]. Noel et al. demonstrated the foremost CH_3_NH_3_SnI_3_ perovskite device administered on a mesoporous TiO_2_ framework. The compound had a bandgap of 1.23 eV. The devices obtained a voltage source of 0.88 V and yielded more than 6% efficiencies.

**Figure 5:**
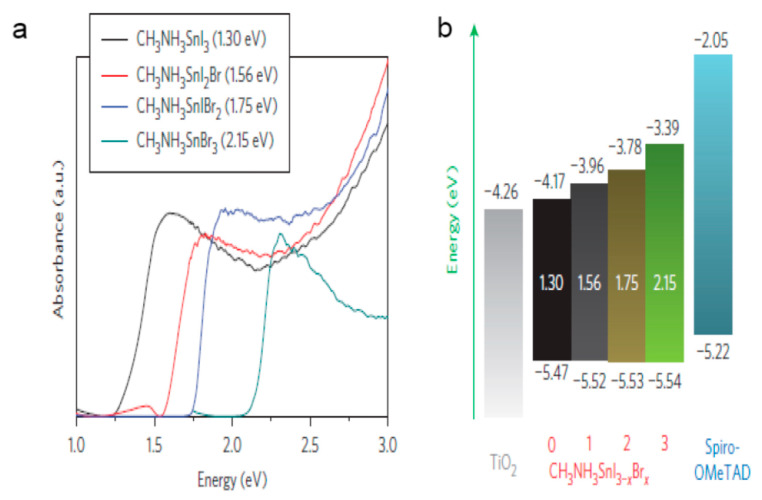
(**a**) UV–visible absorption spectra and (**b**) energy-level diagram of CH_3_NH_3_SnI_3−x_Br_x_ compounds. Reprinted with permission from [54]. Copyright: Springer Nature: Nature Photonics (2014).

Nevertheless, the unstable nature of Sn-based compounds remains a challenge [73]. Hao et al. proceeded to report a lead-free solution administered solid-state photovoltaic device with CH_3_NH_3_SnI_3_ (methylammonium tin iodide) perovskite on organic an hole–transport layer spiro-OMeTAD coupled with mesoporous TiO_2_ support. The CH_3_NH_3_SnI_3_ perovskite material featured a bandgap of 1.3 eV. The devices achieved a voltage source of 0.68 V and yielded an efficiency of 5.23%. Furthermore, bandgap fixing was employed through chemical replacement in the procedure of the CH_3_NH_3_SnI_3−x_Br_x_ solid solution, which remained manageably tailored to cover the visible wavelength region extensively. The CH_3_NH_3_SnI_3−x_Br_x_ perovskite absorber featured a value of 1.75 eV optical bandgap. The solar devices obtained a voltage source of 0.82 V and yielded an efficiency of 5.73%. The open-circuit voltage experienced a tremendous improvement and was attributed to the rise in conduction bandgap by way of growing the Br substance inside CH_3_NH_3_SnI_3−x_Br_x_ perovskite material, as shown in Figure 5 [54].

**Figure 6:**
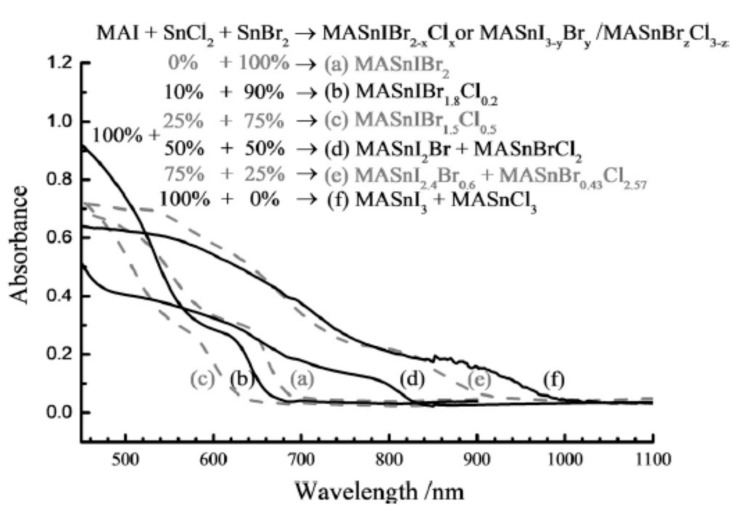
The synthetic method and the absorption spectra of hybrid halide tin perovskites deposited on glass substrates with related proportions of SnCl2/SnBr2: (**a**) 0/100; (**b**) 10/90; (**c**) 25/75; (**d**) 50/50; (**e**)75/25; (**f**)100/0. Reproduced with permission [55]. Copyright: John Wiley and Sons (2017).

Furthermore, Tsai et al. reported the combination of MAI and the SnCl_2_/SnBr_2_ precursors in equal percentages with varied SnCl_2_/SnBr_2_ ratios—0/100, 10/90, 25/75, 50/50, 75/25, and 100/0 to form methylammonium (MA)-mixed tri-halide Sn perovskites, as shown in Figure 6. By starting with 0% SnCl_2_/100% SnBr_2_ ratios, the perovskite material of MASnIBr exhibited absorption at 700 nm by a bandgap of 1.81 eV. Through compounding the quantity of SnCl_2_ from 0% to 25%, the absorption experienced a blueshift, which gave rise to a bandgap with a value of 1.97 eV. Conversely, in the equal ratio of 50% SnCl_2_ /50% SnBr_2_, the absorption spectrum showed a major redshift equal to about 850 nm (Eg = 1.49 eV), further increasing the produced SnCl_2_/SnBr_2_ ratios and furthering the shifts of the spectra with its signal processing close to 1000 nm (Eg = 1.25 eV) at 100% SnCl_2_. The perovskite solar cell device with the proportionate composition of MASnIBr1.8Cl_0.2_ (SnCl_2_ = 10%) exhibited an outstanding device performance of 3.1% PCE, with a voltage source of 0.38 V, a photocurrent of 14.0 mA cm^−2^ and a 57.3% fill factor. Therefore, the experimental work gave credence to the fact that the insignificant amount of Cl within the trihalide perovskite material gave a superior performance of PSCs, which is generally due to the controlled charge recombination, the reduced charge build-up, and the improved exciton lifetime [55].

Recently, Tsarev et al. described the incomplete replacement of monovalent methylammonium (MA) positively charged ions by hydrazinium (HA) ions to enhance the stability of MASnI_3_ films as well as improve their morphology, thereby giving rise to notable build-up concerning the conversion efficiency of solar cell devices. The hydrazinium-loaded layer exhibited immeasurably exclusive stability upon exposure to light beneath an inactive air as likened to the source MASnI_3_ layers, which undertook instant transformation to MA_2_SnI_6_ based the operating conditions. The material photostability significantly improved due to the vigorous inhibition of Sn(II) disproportionation to Sn(IV) and Sn(0) by the incorporating hydrazinium ions. Furthermore, the incorporation of hydrazinium positively influenced the film morphology, whereby there was reduced denseness of holes and pinholes. Moreover, the photovoltaic output performance tremendously improved, whereby the solar cell efficiency increased by starting from 0% for MASnI_3_ to 2.6% for MA_0.8_ HA_0.2_SnI_3_ [56].

**Figure 7:**
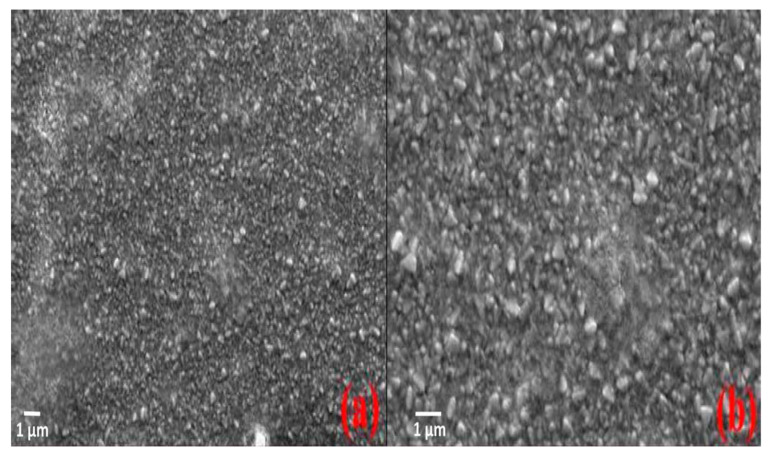
SEM image of (**a**) CH_3_NH_3_SnCl_3_ powder perovskite (**b**) and CH_3_NH_3_SnCl_3_ crystal perovskite. Reproduced with permission [74]. Copyright: Elsevier Ltd. (2017).

Meanwhile, Singh et al. implemented a series of experiments to study the comparative behavior of CH_3_NH_3_Cl powder and crystal as perovskite absorbers for photovoltaic application. The powder perovskite exhibited a bandgap of 2.5 eV, whereas the crystal perovskite showed a bandgap of 2.1 eV. The XRD and SEM result analysis showed structural characterization of perovskite absorber distribution at the titanium dioxide surface and was compared with easily accessible literature. The crystal pattern of the perovskite is confirmed from SEM. The solar device with a PEO-based solid polymer electrolyte obtained an open circuit voltage of over 0.48 V for powder perovskite and 0.60 V for crystal perovskite of CH_3_NH_3_SnCl_3_, while the yielded power conversion efficiency of 0.17% for powder perovskite and 0.55% for crystal perovskite of CH_3_NH_3_SnCl_3_ were recorded at 100 mW/cm^2^ (1 sun condition) processed in the ambient air surrounding. Therefore, the results show that the crystal perovskite is significantly superior to the powder perovskite of CH_3_NH_3_SnCl_3_, as displayed in Figure 7 [74].

By substituting the A cation of CH_3_NH_3_ with HC(NH_2_)_2_ cation, Koh and co-workers identified the FASnI_3_ perovskite film as a light harvester in a solar cell device, which achieved a bandgap with a value of 1.41 eV. By incorporating SnF_2_ on top of mesoporous TiO_2_, the solar device with the capping layer of FASnI_3_achieved a voltage source of 0.238 V as well as efficiency of 2.10% [57]. However, Zhang et al. (2016) reported bandgap engineering by chemical substitution, featuring the composition of FASnI_2_Br obtaining a bandgap with a value of 1.68 eV. By combining C_60_ by way of electron-transport coating and a MoO_x_ film-based inorganic substance as a novel kind of free hole-transport coating with lead-free FASnI_2_Br perovskite, the perovskite of the Sn-based solar cell obtained a voltage source of 0.467 V and yielded an efficiency of 1.72% [75].

Moreover, Ke at al. demonstrated a novel kind of tin-centered perovskite absorber whereby ethylenediammonium (en) was incorporated into formamidinium (FA), resulting in the formation of new types of cavity 3D structures of {en}FASnI_3_ perovskite. It was observed that the incorporation of ethylenediammonium (en) into the A cation structure achieved an evident rise in the bandgap deprived of the necessities for solid solutions, material stableness, and enhanced photoelectric qualities of the formulated Sn-based absorbers. The superlative attainment of the 3D perovskite absorber by way of 10% en loading could be attributed to 1.5 eV as an ideal bandgap. Upon capping with an agent of {10% en} into the FASnI_3_ compound on a thin bedrock of poly[bis(4-phenyl)(2,4,6-trimethylphenyl) amine] (PTAA) as a hole-transporting layer as well as a 1 mm-thick substrate of mesoporous TiO_2_, the solar cell device obtained 7.14% PCE through a voltage source of 0.480 V, a photocurrent of 22.54 mA cm^−2^, and a fill factor of 65.96% [58]. On the other hand, Kayesh and co-workers demonstrated the incorporation of measured hydrazinium chloride (N_2_H_5_Cl) substance into an unmixed precursor solvent entity to formulate FASnI_3_ perovskite layers, which achieved a bandgap with a value of 1.37 eV. The outstanding incorporation of N_2_H_5_Cl resulted in decreased concentration of Sn^4+^ substance by 20% in the FASnI_3_ layer, thereby ensured the inhibition of the carrier recombination and the possibility to create pinhole-free uniform coverage on the substrates. The noteworthy enhancements reached in the Sn-based PSC coupled with FASnI3 film exhibited up to 5.4% power conversion efficiency and a major rise in the voltage source of 0.455 V [59].

**Figure 8:**
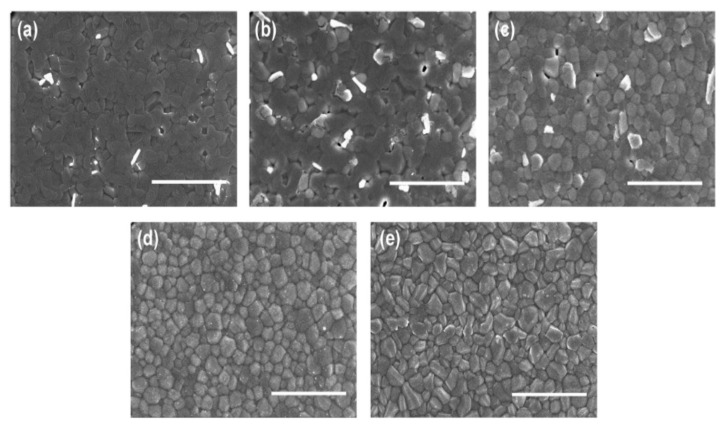
SEM images of (**a**) MASnI_3_, (**b**) (formamidinium (FA))_0.25_(methylammonium (MA))_0.75_SnI_3_, (**c**) (FA)_0.50_(MA)_0.50_SnI_3_, (**d**) (FA)_0.75_(MA)_0.25_SnI_3_, and (**e**) FASnI_3_ films deposited on indium tin oxide (ITO)/ PSS poly(3,4-ethylenedioxythiophene) poly(styrenesulfonate) (PEDOT): PSS substrates (scale bar: 3.0 µm). Reproduced with permission [37]. Copyright: WILEY-VCH Verlag GmbH & Co. KGaA, Weinheim (2017).

Through varying the percentage of both contents, namely, formamidinium (FA) and methylammonium (MA) positively charged ions, Zhao et al. reported a tin-based hybrid compound of (FA)_x_(MA)_1−x_SnI_3_ (FA = NH_2_CH = NH_2_^+^, MA = CH_3_NH_3_^+^), which was designed with the aim of developing inverted perovskite fabricated devices. The bare MASnI_3_ film showed a more continuous distribution without significant traces of grain boundaries. As the content of FA was enhanced, the grain boundaries slowly became obvious, and the FASnI_3_ film showed crystal grains with sharp edges and clear boundaries. Some white grains were found in the films at lower FA content (x = 0.00, 0.25, and 0.50), which may be attributed to the phase partition caused by SnF_2_. These films also showed partial coverage through a small pinhole, which explained the low fill factor (FF) and the slightly lower average short-circuit current (Jsc) of their corresponding devices. However, the film morphology was greatly improved at higher FA contents (x = 0.75 and 1.00), which displayed whole coverage coupled with no obvious phase partition, thereby leading to superior system performance. The best performing (FA)_0.75_(MA)_0.25_SnI_3_ lead-free perovskite material obtained a bandgap of 1.33eV. The PSCs device with (FA)_0.75_(MA)_0.25_SnI_3_ through incorporating 10 mol% SnF_2_ additive exhibited 8.12% PCE and a better voltage of 0.61 V, which originated from better-quality pinhole-free perovskite layer morphology and hinders recombination activity in the system, as shown in Figure 8. Therefore, composition engineering of the requisite compound remains a unique technique for obtaining superior Voc along with PCE aimed at the stable performance of Sn-based perovskite solar cells [37].

Similarly, Gao and co-workers reported a structural regulation strategy through the insertion of cesium cation (Cs^+^) into the composition of FASnI_3_ for the formation of 3D Sn-faced lead-free Cs_x_FA_1−x_SnI_3_. The inverted planar perovskite solar cell (PSC) constructed with Cs_0.08_FA_0.92_SnI_3_ showed 6.08% PCE and a significant voltage source of 0.44 V. Through their experimental and theoretical work, Gao et al. improved the spatial regularity, suppressed the loss of the electron in regard to Sn^2+^ and enhanced the thermodynamically structural solidity of FASnI_3_ by incorporating the Cs^+^ ion [60]. However, Shao et al. had a breakthrough in reporting a PCE as high as 9.0% through a voltage source of 0.525 V for formamidinium tin iodide (FASnI_3_) perovskite films featuring inside a planar p–i–n device assembly. The best performing perovskite films were synthesized by mixing both contents of a very little quantity (0.08 m) of 2D Sn-perovskite substrate and 0.92 m of 3D Sn-perovskite to induce top-quality crystallinity having distinct coordination of the 3D FASnI_3_ granules. The chemical platform for increased ordering and packing of crystal planes enhanced the toughness and reliability of the perovskite composition, which helped to restrain the configuration of tin vacancies and cause an overall background carrier density over the material. Thus, improved solar cell performance was a result of the great level of crystallinity and superior crystal coordination [61].

Using cesium (Cs) as the A cation in a 3D Sn-based perovskite, Chung et al. explained the synthesis of cesium tin iodide (CsSnI_3_) perovskite featuring 1.3 eV for the optical bandgap [76]. Moreover, Chen et al. demonstrated the preparation of CsSnI_3_, exhibiting an optical value of 1.3 eV as a bandgap. The Schottky solar cells with CsSnI_3_ obtained an efficiency value of 0.9% and a voltage source of 0.42V [77]. Subsequently, Kumar et al. illustrated the experiment of minimum temperature (70 °C) processed (CsSnI_3_) to develop solar cells exhibiting photocurrents beyond 22 mA/cm^2^ coupled with a spectral response increasing in the direction of 950 nm. The CsSnI_3_ perovskite possessed a value of 1.3 eV as an optical bandgap. Since CsSnI_3_ was being used as a photoabsorber, and with the ability to be disposed to form inherent defects linked with Sn cation vacancies that produce metallic conductivity, Kumar and co-workers demonstrated the inclusion of SnF_2_ as a stabilizer to control the metallic conductivity. The best performing CsSnI_3_ perovskite absorber with 20% SnF_2_ loading was observed upon its use on a solar device, producing a 2.02% PCE and 0.24 V as an open circuit voltage [62].

Besides, Sabba et al. rationalized the combination of various compositions of CsSnI_3−x_Br_x_ by also specifically partially replacing bromine into bare CsSnI_3_ as a follow up to the previous report by Kumar and co-workers in a bid to further study the outcome of replacing bromine into the bare substance of CsSnI_3_ in the existence of SnF_2_. Amidst the different structures being synthesized, CsSnI_2.9_Br_0.1_ produced superlative performing results with a voltage source of 0.222 V, a photocurrent of 24.16 mA/cm^2^ as well as a fill factor with a value of 0.33, thereby indicating 1.76% conversion efficiency. By gradually replacing I^−^ with Br−, the perovskite absorber registered an increase in the optical bandgap through 38.5% alongside a blue shift in the absorption spectra. The favorable outcome of Br content inclusion for voltage enhancement was obvious for the CsSnI_3_ structure even in the absence of SnF_2_. The voltage enhancement was attributed to the reduction in Sn defects based on its missing atoms in its lattice sites, wherein it is revealed through the inferior carrier concentration or charge carrier densities of 10^15^ cm^−3^, as well as an extraordinary opposition to the mechanism of charge recombination in the instance of the Br-loaded CsSnI_3−x_Br_x_ compound. The outstanding current densities were enhanced greatly due to the incorporation of SnF_2_ into the CsSnI_3−x_Br_x_ compound [63].

Recently, Zhang et al. for the first time reported the chemical alloying substance, specifically, cobaltocene (by the formula of Co(C_5_H_5_)_2_), possessing an intense electron releasing ability as an additive to affect stability as well as influence the electrical property of the CsSnI_3_ perovskite substance for photovoltaic applications. Cobaltocene (Co(C_5_H_5_)_2_) is a commonly used one-electron reducing agent that can simply transfer an “extra” electron from the metal cobalt to form an 18-electron positively charged ion with great stableness. The CsSnI_3_ perovskite material is attributed to have chemical diversity and complex conformational space, proffering extremely promising adjustable composition in relation to chemical redox molecules. As a result of the outstanding attributes of the CsSnI_3_ perovskite material, an electron-rich environment provided by incorporating cobaltocene (Co(C_5_H_5_)_2_) offered the proficiency of inhibiting Sn_2_^+^ oxidization and reducing the trap density when compared to the plain CsSnI_3_. A solar cell device with the best performing CsSnI_3_ of 1% (Co(C_5_H_5_)_2_) loaded achieved a voltage source of 0.46 V and yielded a 3.0% conversion efficiency [64]. Therefore, the novel study provided an effective method for assembling active and steady solar cell devices by integrating donor elements to inhibit Sn^2+^ oxidation.

Conversely, Gupta et al. explained the synthesis of cesium tin bromide (CsSnBr_3_) perovskite material by way of an effective light harvester for the solar cells, exhibiting a direct bandgap with a value of 1.75 eV. The best performing solar cells with CsSnBr_3_ (with 20 mol% SnF_2_ additive) showed 2.1% conversion efficiency by way of J_SC_, a photocurrent of around 9 mA cm^−2^, a V_OC_ source of 0.41 V, and fill factor with a value of 58% underneath 1 sun (100 mW cm^−2^) illumination when compared to the photovoltaic performance of solar cells with bare CsSnBr_3_. As stated earlier in previous sections, the addition of tin fluoride (SnF_2_) remained focused on the procurement of positive device operating results based on its primary function to counteract traps by way of occupying lattice spaces for the reduction of the surroundings carrier density. Therefore, it becomes clear that by way of appropriate encapsulation, Sn-based solar cells may grow into valuable mutually separate building blocks or as a part of larger cell structures [65].

### 2.3. Ge-Based 3D Perovskite Absorbers (AGeX_3_)

Ge belongs to the carbon family of group IV in the periodic table, similar to Sn and Pb elements. It is an element that is being studied to show potential responsibility for the actualization of perovskite solar cell applications. When compared to Pb^2+^, Ge^2+^ has the characteristics of greater electronegativity, a new covalent property, and an ionic radius (73 pm) seen to be lesser than that of Pb (119 pm). Theoretical studies have attested to the potency of using germanium halide perovskites for solar cell applications, but they have been rarely investigated experimentally. Based on theoretical and experimental studies, Stoumpos et al. reported the effects of the mixed organic/inorganic germanium perovskite materials with formula AGeI3 (A = Cs, organic cation) experimentally. Through CsGeI_3_ as the model composition, the following 3D compounds were prepared: methylammonium (CH_3_NH_3_GeI_3_), formamidinium (HC(NH_2_)_2_GeI_3_), acetamidinium (CH_3_C(NH_2_)_2_GeI_3_), and guanidinium (C(NH_2_)_3_GeI_3_). The 3D perovskite materials exhibited direct bandgaps of 1.6 eV for CH_3_NH_3_GeI_3_, 1.9 eV for HC(NH_2_)_2_GeI_3_, 2.2 eV for CH_3_C(NH_2_)_2_GeI_3_, and 2.5 eV for C(NH_2_)_3_GeI_3_. Based on structural grounds, the consequences of Cs substitution with larger cations of the organic compounds will activate the stereochemical representation of the 4s^2^ “inert pair” (likewise known as lone pair activation) which thereby activates the alteration of the band formation, resulting in a great enlarging of the bandgap [78].

Furthermore, Krishnamoorthy et al. synthesized three halide perovskite compounds with the formulation of AGeI_3_ (A=Cs, CH_3_NH_3_, or HC(NH_2_)_2_). The compounds remained stable until 150 °C, and possess bandgaps linked to the A-site positively charged ion size. The bandgaps exhibited by the materials were 1.63, 2.0, and 2.35 eV for CsGeI_3_, MAGeI_3_, and FAGeI_3_, respectively. CsGeI_3_ and MAGeI_3_ were observed to have relatively smooth morphology, while that of FAGeI_3_ was very poor. Thus, the device-fabricated solar cells with the germanium iodide perovskites of both CsGeI_3_ and MAGeI_3_ using compact and mesoporous TiO_2_ and Spiro-OMeTAD as electron- and hole-selective contacts exhibited a PCE of 0.11% and a voltage source of 0.074 V for CsGeI_3_ as well as a 0.20% PCE and voltage source of 0.15 V for MAGeI_3_.

Based on the poor film quality of FAGeI_3_, solar cells did not show any photoelectric properties. The poor performance of the devices could be attributed to Ge^4+^ formation by oxidation. Overall, the study showed a strong potential for Ge-based halide perovskite compounds in photovoltaic applications [66]. Recently, Kopacic et al. explained the synthesis of germanium halide perovskites from GeI_2_, MAI, and MABr, respectively, by using DMF as a solvent for the precursor solution. Kopacic et al. showed that chemical composition plays a crucial role in improving material stability and performing capacity of the germanium perovskite compounds when adapted to function in a fabricated solar cell. Hence, Kopacic et al. observed excellent material stability and unique operating capacity through chemical composition by incorporating bromide ions into the matrix of methylammonium germanium iodide perovskite. The planar p–i–n solar cells with the best-performing MAGeI_2.7_Br_0.3_ obtained a value of 0.57% PCE. Based on this study, it was shown that the bromide substance in methylammonium germanium halide perovskites shows a key function in the performance of the photovoltaic devices [67].

## 3. Double Perovskite Absorbers (A_2_BB′X_6_)

**Figure 9:**
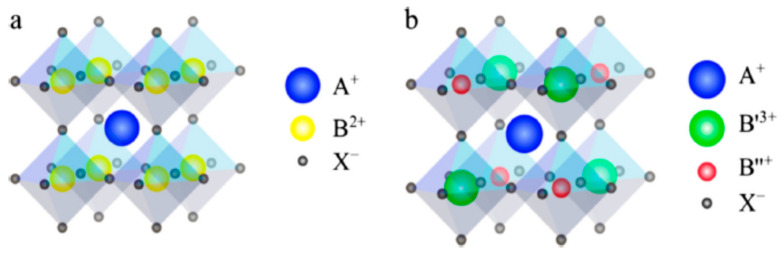
The structures of the conventional ABX_3_ (**a**) and double A^I^_2_B^I^B^III^X_6_ (**b**) perovskite. Reproduced with permission [80]. Copyright: Elsevier Ltd. (2018).

The double perovskite, converted to a quaternary A^I^_2_B^I^B^III^X_6_ formula, is a neighboring derivative of the ABX_3_ single hybrid halide perovskites. Their derivatives are composed of metallic halide octahedra B^I^X_6_ or B^III^X_6_ units that assign intersections among six adjacent octahedra having dissimilar ions to develop 3D lattice, as shown in Figure 9. The monovalent and trivalent metal ions coexisting together are enshrined in the crystal composition of a standard double perovskite. The two distinct monovalent and trivalent metal ions that periodically substitute for the toxic Pb^2+^ in a single perovskite have copious elementary combinations that furnish extensive alternatives aimed at achieving preferred qualities [79]. Herein, a summary of device performance on selected metal halide double perovskite solar cells is listed in Table 2. Meanwhile, an overview of metal halide double perovskite on their material compositions, bandgaps, and morphological properties alongside synthetic methods is listed in Table 3.

Due to the investigation for more lead-free perovskite materials, a novel class of double perovskite materials has been produced. Slavney and coworkers replaced Ag^+^ and Bi^3+^ for toxic Pb^2+^ taken with the perovskite network in Cs_2_AgBiBr_6_. The perovskite material showed a direct bandgap value of 1.95 eV as well as basic photoluminescence life of about 660 ns, which is suited for photovoltaic applications. The photoluminescence (PL) decay curve of Cs_2_AgBiBr_6_ showed a great defect tolerance based on the contrast involving single crystal and powder. Moreover, it showed superfluous heat and moisture stability when likened to (MA)PbI_3_ [94]. McClure et al. reported the preparation of Cs_2_AgBiBr_6_ and Cs_2_AgBiCl_6_ double perovskites through both the solid and synthetic solution pathways. The relative diffuse reflectance measurements showed bandgaps with a value of 2.19 eV for Cs_2_AgBiBr_6_ and a bandgap with a value of 2.77 eV for Cs_2_AgBiCl_6_. The density of state calculation for the bandgap indicated that transition from the valance to the conduction band takes place from the occupied halogen 3p/4p orbitals to antibonding Ag 5s and Bi 6p orbitals. The presence of the Ag 4d orbitals reduces the bandgap and interacts with the notable field of 3p/4p orbitals situated in the halide ion to ensure modification of the valance band, thereby producing an indirect bandgap. The two composites are stable once opened to the atmosphere [95].

Through theoretical study and experimental synthesis, Volonakis et al. reported photosensitive features in the group of double perovskite A_2_B′B″X_6_ with A = Cs, B′ = Bi, Sb, B″ = Cu, Ag, Au and X = Cl, Br, I. Theoretical study envisaged that all hybrids possess indirect bandgaps coupled with the projected approach for tuning them into direct bandgaps. Furthermore, the double perovskite of Cs_2_AgBiCl_6_ was successfully synthesized, and it was shown to belong to the fm3m space assembly and consist of BiCl_6_ and AgCl_6_ octahedra, which are fluctuating in a rock-salt-face-centered structure. The hybrid revealed an indirect bandgap with a value of 2.2 eV [85]. Greul et al. synthesized and reported Cs_2_AgBiBr_6_ film being annealed at 250 °C with a 2.5% conversion efficiency and a voltage source surpassing 1 V. This stands presently as the maximum recorded voltage source for bismuth halide perovskite materials, thereby serving as a potential light-absorbing double perovskite material [96].

Even though halide double perovskites have gained attention due to their composition of lesser toxicity elements, stability in the air, and long carrier life, there are still challenges most double perovskites, including Cs_2_AgBiBr_6_, have faced due to their wide bandgaps that limit photoconversion efficiencies. Hence, Li et al., through a solution-based route, effectively synthesized phase-pure Cs_2_AgSbBr_6_ thin films, as well as Cs_2_Ag(Sb_x_Bi_1−x_)Br_6_ with the mixing alloys of parameter x continuously changing over the complete composition extent (x between 0.5 and 0.9). Through this novel route, Li et al. reduced the 2.25 eV bandgap of Cs_2_AgBiBr_6_ and 2.18 eV of Cs_2_AgSbBr_6_ to as low as 2.08 eV of Cs_2_Ag(Sb_x_Bi_1−x_)Br, thereby proving the fittingness of double perovskites for photovoltaic and photocatalytic applications [86]. By way of implementing a crystal engineering approach which offered to transform the standard double perovskites of Cs_2_AgBiBr_6_ by simply influencing the gradual crystal change in temperature and speed, Klarbring et al. attained the minimum disclosed bandgap of lead-free double perovskite Cs_2_AgBiBr_6_ to appreciably drop in bandgap value by around 0.26 eV, thereby attaining the lowest described bandgap of 1.72 eV for Cs_2_AgBiBr_6_ in ambient settings, whereby bandgap reduction was validated by both electronic absorption and photoluminescence magnitudes [87].

Due to the morphology engineering of Cs_2_AgBiBr_6_, Gao and co-workers focused on improving the film-forming ability of the perovskite, because it is known that perovskite film with high surface coverage and less defects is influential to the rapid transportation of both electrons and holes, including having an evident effect on device stability and best performing ability [81,97]. Hence, the results showed Cs_2_AgBiBr_6_ film morphology with granules composed of quality crystals that were obtained via the method of anti-solvent treatment annealed at high temperatures, thereby exhibiting an indirect bandgap value of 1.91 eV. Conversion efficiency with a value of 2.2%, a voltage source of 1.01 V, a photocurrent of 3.19 mA/cm^2^ and a fill factor with a value of 69.2% were exhibited via an inverted planar heterojunction solar cell device with a Cs_2_AgBiBr_6_ layer. The device established material stability with no hysteresis [81]. Meanwhile, Wu et al. reported 1.44% power conversion efficiency with a voltage source of 1.04 V, a photocurrent of 1.78 mA/cm^2^, and a fill factor with a value of 78%, as attained in the Cs_2_AgBiBr_6_ film being annealed at 250 °C [98]. More importantly, the film shows superior thermal and ambient stability, thereby revealing the potential for lead-free perovskite solar cells. The figure shows SEM and XRD spectra. SEM of Cs_2_AgBiBr_6_ film annealed at 250 °C shows smooth morphology which influenced the rapid transportation of both electrons and holes [81]. Using the first planar structure solar device based on double perovskite Cs_2_AgBiBr_6_, Ning et al. demonstrated, by preparing Cs_2_AgBiBr_6_ layers that are made of high-level crystal property granules with diameters equivalent to the layer texture, a reduction in the granule periphery length and carrier recombination. The Cs_2_AgBiBr_6_ layers show elongated electron–hole diffusion lengths of more than 100 nm, thereby supporting the assembly of planar construction double perovskite solar cells. The resultant solar cells built on planar TiO_2_ exhibited over 1% typical power conversion efficiency [99].

Bandgap engineering provides an important technique for narrowing the large indirect bandgap, thereby targeting photovoltaic performance. Using Cs_2_AgBiBr_6_ as a high-value target compound, bandgap engineering through alloying In^III^/Sb^III^ of has been duly recorded. Du et al. reported the fixing of bandgaps concerning Cs_2_Ag(Bi_1−x_M_x_) Br_6_ (M = In^III^/Sb^III^) by introducing In^III^/Sb^III^ to take up to 75% of In^III^, which resulted in an enlarged bandgap equal to 37.5% Sb^III^ with a diminished bandgap, thereby supporting bandgap modulation of about 0.41 eV throughout the incorporation of the dual metals through a minimum 1.8 eV bandgap on behalf of Cs_2_Ag(Bi_1−x_ M_x_)Br_6_. The different atomic configurations for the two metals (In/Sb) are responsible for the difference in bandgaps shifting directions [100]. Meanwhile, Liu et al. demonstrated bandgap tailoring of Cs_2_AgBiBr_6_ by incorporating only Sb to replace 75% content of Bi by the use of a solution-processed approach in a solvent medium of dimethyl sulfoxide at 180 °C. The Sb substitution drastically reduced the bandgap of Cs_2_Ag(Sb_x_Bi_1−x_)Br_6_ at a value of 2.22 eV when x = 0 to a value of 1.97 eV when x = 0.75. The effective bandgap reduction through the simple solution might speed up the enhancement of Cs_2_AgBiBr_6_ double perovskite for photovoltaic applications [101].

Furthermore, Wei and co-workers described the preparation of double perovskite Cs_2_AgSbBr_6_ with the aim of evaluating and maximizing its utilization. Using hydrothermal methods, a black Cs_2_AgSbBr_6_ was successfully prepared with a value of 1.64 eV to support a low bandgap. However, the compound experienced a color change from black to brown due to the occurrence of charge transfers from Sb^3+^ to Sb^5+^. A working device with Cs_2_AgSbBr_6_ as a solar absorber on fluorine-doped tin oxide (FTO)/TiO_2_/perovskites/spiroOMeTAD/Au architecture was fabricated and exhibited a 0.01% efficiency, a voltage source of 353.29 mV, a short circuit current of 0.08 mA cm^−2^ and a fill factor with a value of 35.9% on perfect films without antisolvent preparation [88]

Through the rich substitutional chemistry attributes of hybrid lead-free halide double perovskite for providing novel classes of perovskite materials [99], Volonakis, et al. reported bandgap engineering via compositional engineering of Cs_2_AgBiBr_6_ by introducing In and Cl or Br to substitute for Bi and Br, respectively, to achieve both double perovskites of Cs_2_AgInCl_6_ and Cs_2_AgInBr_6_. The double perovskite of Cs_2_AgInCl_6_ attained a successful synthetic outcome through a measured 3.3 eV bandgap, and X-ray diffraction produced a configuration with the space group Fm3m. Hence, the synthesis of mixed halides has created more possibilities intended for the growth of double perovskite and tunable bandgaps [102]. Meanwhile, Zhou et al. designed and reported bandgap engineering via compositional fixing of Cs_2_AgBiBr_6_ by presenting In and Cl to replace Bi and Br, respectively. Due to the design for hydrothermal synthesis of Cs_2_AgInCl_6_ in regard to the growth of single crystals, the perovskite material crystallized Cs2AgInCl_6_ into a single crystal via the chemical composition of alternating octahedra of [AgCl_6_] and [InCl_6_] in the salt composition. Cs_2_AgInCl_6_ experimentally obtained a 3.23 eV direct bandgap and theoretically obtained a 3.33 eV bandgap [103]. Besides, the perovskite material showed exceptional moisture, light, and heat stability, which indicates a huge possibility for photovoltaic application through further bandgap engineering [102]. Dahl et al. carried out the preparation of nanocrystals of Cs_2_AgInCl_6_ and Cs_2_AgSbCl_6_ using the colloidal preparation technique of introducing acyl halides within the ambient of the surrounding temperatures. The two compounds displayed bandgaps wherein Cs_2_AgSbCl_6_ showed a value of 2.57 ± 0.05 eV as an indirect bandgap, and Cs_2_AgInCl_6_ displayed a value of 3.57 ± 0.03 eV for a direct bandgap. Based on degradation assessment, Cs_2_AgInCl_6_ showed a decrease in material stability over Cs_2_AgBiCl_6_ to Cs_2_AgSbCl_6_ [90]. However, Zhou et al. utilized the colloidal preparation technique through a modified one-pot hot injection system to prepare a Cu(I)−Sb-based double perovskite nanocrystal of (Cs_2_CuSbCl_6_ NCs) with the smallest bandgap of 1.66 eV when compared to other lead-free double perovskites nanocrystals. Due to the compound’s unique stability to ambient air, the outstanding properties of the compound could make it a suitable light-absorbing material for photovoltaic utilization [91].

However, Karmakar et al. reported bandgap engineering via the compositional engineering of Cs_2_AgBiBr_6_. By incorporating Sb and Cl to replace Bi and Br, respectively, with doping of Cu^2+^, Cs_2_SbAgCl_6_ was formed, whereby the perovskite materials exhibited an effective shift in their bands from around a value of 2.6 eV (parent Cs_2_SbAgCl_6_) to around a value of 1 eV (Cu^2+^-doped Cs_2_SbAgCl_6_). The patterns of XRD for the Cu^2+^-doped Cs_2_SbAgCl_6_ polycrystalline materials indicated an extended range of crystallinity through non-uniform microstrain in the crystal lattice. The perovskite materials indicated thermal and moisture stability based on a comprehensive stress analysis that was conducted on the material for 365 days [82]. However, Zhang et al. designed and reported bandgap fixing for Cs_2_AgBiBr_6_ by presenting Na and I to replace Ag and Br, respectively, thereby giving rise to new and extremely stable Cs_2_NaBiI_6_ compounds. The new material has a value of 1.66 eV for a low bandgap and exhibited high stability when exposed to the surrounding air. The fabricated device constructed on Cs_2_NaBiI_6_ exhibited 0.42% conversion efficiency with a voltage source of 0.47 V, a photocurrent value of J_SC_ = 1.99 mA/cm^2^, and a 44% fill factor, while the fabricated Cs_2_NaBiI_6_ as light absorber film revealed great stableness and reproducibility [104]. Moreover, Peedikakkandy et al. analyzed the fact that the compound Cs_3_Bi_2_I_9_ of iodobismuth ternary perovskites has recorded some achievements, but a wide bandgap and lower structure dimensions are its visible limitations. As such, Peedikakkandy et al. altered the broad bandgap of Cs_3_Bi_2_I_9_ composition to a small bandgap of double perovskite of Cs_2_NaBiI_6_ by Na_2_S inclusion, thereby presenting a near-to-finest bandgap of ∼1.5 eV. The sodium ion in Na_2_S plays a major role in replacing a trivalent bismuth ion of the Cs_3_Bi_2_I_9_ composition and forces the conversion to a double perovskite formation of (Cs_2_NaBiI_6_), while the divalent sulfur ion tends to occupy the crystal voids and tailor the bandgap. The XRD analysis of the altered Cs_2_NaBiI_6_ showed a great level of crystallinity, thereby giving a suitable morphology to support the utilization of its films for solar cell devices [92]. Alternatively, Cao et al. used the solid-state reaction method and the hydrothermal method by incorporating VCl_3_ as the vanadium source to prepare a red crystal halide double perovskite of Cs_2_NaVCl_6_ by way of reaching a bandgap with a value of 2.64 eV. Moreover, the material exhibited extraordinary attributes by showcasing two-fold strong absorption bands at 558 and 900 nm, which can be considered for photosensitization [89].

The introduction of gold as an element into the composition of double perovskites is of paramount importance due to its electronic properties. For the preparation of mixed gold halide double perovskites, Ghosh et al. utilized a solution-processed route to produce a tetragonal crystal of (MA)_2_Au_2_X_6_, (X = Br, I) double perovskites with an ideal bandgap of around 1.0 eV less than traditional halide-oriented compounds of double perovskites. Even though the compound was found to be hygroscopic, its single crystals and the thin film showed no degradation, thereby indicating superior material stability. The double perovskite showed photoresponse joined by small confined density, thereby indicating its possibility in PV utilization [93].

### Ordered-Vacancy Double Perovskite Absorbers (A_2_BX_6_)

Ordered-vacancy double perovskites converted to the typical formula (A_2_BX_6_) are close derivatives of the ABX_3_ single metal halide perovskites, whereby their derivatives are constituted of a face-centered network of closely secluded [BX_6_] components through A-site cations inhabiting the cuboctahedral spaces [22,109]. Due to the quest for the production of chemical compositions meant for solar cell deployment, Lee et al. introduced a new group of atomic iodosalt compositions of Cs_2_SnI_6_ whereby Sn stays as a cutting edge with +4 oxidation state, thus fashioning the material to be stable in atmospheric conditions. Using Cs_2_SnI_6_ as a hole-transporting agent, a mesoporous TiO_2_ film dye-sensitized solar cell (DSSC) was successfully fabricated in ambient air, which delivered 4.7% of power conversion efficiency [110].

Furthermore, to overcome the synthetic challenges in solution processing of Cs_2_SnI_6_ and to boost optimal material performance, Lee et al. established a two-step solution technique. In step 1, a pure crystal-like layer was produced with the aid of SnI solution, while in step 2, a new series of Cs_2_SnI_6−x_Br_x_ films was established on comprehensive structural, electrical, and optical analysis. These air-steady molecular semiconducting iodosalts of Cs_2_SnI_6−x_Br_x_ provide values of around 1.2 eV to 2.9 eV as the desired bandgaps within the range of x where x < 3. By employing the solar cell device with Cs_2_SnI_6−x_Br_x_ films, it showed a 2.1% power conversion efficiency based on the case where x = 2 of the requisite compound [83]. However, Saparoy et al. developed and reported a vacuum-based deposition technique aimed at synthesizing a single-phase film of Cs_2_SnI_6_, wherein it yielded a 1.6 eV direct bandgap. Cs_2_SnI_6_ was used for all fabrication processes in the air-surrounding environment, indicating a promising future of inexpensive, clean, safe, and stable solar cell productions [111]. Using the hydrothermal method, Han and co-workers demonstrated the formulation of a double perovskite devoid of lead such as Cs_2_SnX_6_ (X = I and Br) powders. As one of the constitute of the perovskites, half of the Sn locations are occupied by vacancies and maintain their 4+ oxidation state. The bandgaps of Cs_2_SnI_6_ and Cs_2_SnBr_6_ were established to be 1.84 and 1.42 eV, respectively, thereby showcasing these perovskites as futuristic-based perovskites for PV utilization [105].

Furthermore, Karim et al. prepared solid solutions of mixed A_2_SnX_6_ compounds to study their photophysical and electronic attributes, whereby Cs_2_SnCl_6_, Cs_2_SnBr_6_, and Cs_2_SnI_6_ exhibited optical bandgaps with values of around 4.89 eV, 3.23, and 1.35 eV, respectively [106]. Meanwhile, Schwartz et al. demonstrated the preparation of Cs_2_PtI_6_ crystal composition with a bandgap value of 1.4 eV. An assembly of planar superstrate n–i–p solar cells administered with the configuration of F: SnO2/CdS/ Cs_2_PtI_6_/carbon/Cu showed an efficiency of 13.88% with low Voc shortfall [107].

Despite the isolated octahedral units, ordered-vacancy double perovskite Cs_2_SnI_6_ has recently been studied for photovoltaic application due to the space-efficient composition of the iodide network that delivers electronic distribution. Maughan et al. furthered this research by using the solid solution method to prepare Cs_2_SnI_6_ from the formulation of Cs_2_Sn_1−x_TexI_6_, and by substitution of tin with tellurium the compound Cs_2_TeI_6_ perovskite was formed. The Cs_2_SnI_6_ perovskite material exhibited a 1.25 eV optical bandgap, whereas Cs_2_TeI_6_ displayed a 1.59 eV optical bandgap. However, the existence of exciton-like attributes close to the absorption threshold ascending from the Te(IV) 5s^2^ electron made the establishment of the optical gap for Cs_2_TeI_6_ problematic. The tightly packed anionic network and the interface concerning the B-site ions and the halide within these materials are solely responsible for their general structure−property relationships. The arrangement and uniformity of the conduction band due to the valence bands that are held by the I 5p orbitals have caused major variation concerning the electronic peak compositions of Cs_2_SnI_6_ and Cs_2_TeI_6_.

Moreover, the intensification in the covalent bonds of the [TeI_6_] octahedral constituents corresponding to that of [SnI_6_] was due to the enlarged Pauling electronegativity of Te^4+^ (χ = 2.1) correlated to that of Sn^4+^ (χ = 1.8). However, there was no measurable PL intensity on cold-pressed polycrystalline pellets of Cs_2_SnI_6_ and Cs_2_TeI_6_. From the findings, experimental investigation offers a background view to comprehend composition−property interactions of cutting-edge light-harvesting compounds [109].

For this reason, that lattice dynamics and structural instabilities strongly control the electronic properties of double perovskite halide semiconductors. Maughan et al. further studied the Rb_2_SnI_6_ vacancy-ordered double perovskite. By substituting Cs^+^ ion with the lesser Rb^+^ ion inside the Cs_2_SnI_6_ compound, the formation of Rb_2_SnI_6_ was accompanied by tremendous changes in structural and electronic behavior. Based on synchrotron powder diffraction analysis, Rb_2_SnI_6_ took on the tetragonal lattice with omitted Sn ions or atoms that were orderly arranged in the double perovskite composition at ambient temperature and went through a stage conversion to a lower-symmetry monoclinic configuration when cooled, being identified through collective octahedral slanting of the [SnI_6_] octahedra. At all given temperatures, the studies on X-ray and the neutron pair scattering function suggested that the confined complex surroundings of Rb_2_SnI_6_ were coherent with monoclinic composition. This finding may well be justified from the bond valence study, which indicated that Rb^+^ ion bonding is enhanced in the monoclinic composition. However, this was not so in the case of Cs_2_SnI_6_, in that at all given temperatures take on the cubic vacancy-ordered double perovskite compositions [22].

Based on the solution-processed approach being supported by reaction mixture oxidation of Pd^2+^ to Pd^4+^, Sakai and co-workers reported a new-type of lead-free material, i.e., hexabromopalladate(IV) (Cs_2_PdBr_6_). The Cs_2_PdBr_6_ formed into a cubic network with the space group Fm3m, providing evidence of unique composition and chemical stability. The optical analysis confirmed that the multi-layered structure exhibited a bandgap value of 1.6 eV. Cs_2_PdBr_6_ showed a photoresponse based on an assembly of indium tin oxide (ITO)/Cs_2_PdBr_6_/Ag, thereby indicating its suitability for utilization in optoelectronic and photovoltaic applications [112]. Meanwhile, Chen and co-workers operated a two-step vapor deposition technique for the formation of titanium-based vacancy-ordered halide double perovskites, such as the synthesis of cesium titanium(IV) bromide (Cs_2_TiBr_6_). The Cs_2_TiBr_6_ films showed a favorable bandgap value of 1.8 eV, elongated and well-adjusted carrier diffusion lengths greater than 100 nm, appropriate energy levels, and environmental stability. Cs_2_TiBr_6_ thin films of a perovskite solar cell device exhibited power conversion efficiency with a value of 3.3%. By alloying with iodide or chlorine, it was foreseen that composition engineering of Cs_2_TiBr_6_ thin films would provide a group of titanium-based double halide perovskites along with a controlled bandgap and properties for a wide range of optoelectronic applications [108].

Ju et al. experimentally prepared a family of titanium-based ordered-vacancy double halide perovskites through composition engineering by alloying with iodide or chlorine of Cs_2_TiBr_6_ thin films. Following a sequence (x = 0, 2, 4, 6) of Cs_2_TiIxBr_6−x_, Cs_2_TiI_6_ and Cs_2_TiBr_6_, as end members, they were synthesized through the melt-crystallization method. Based on optical analysis, the prototypical members of the Ti-based perovskite extraction processes such as Cs_2_TiIxBr_6−x_ showed tailored bandgaps linked to the 1.38 and 1.78 eV standard values for photovoltaic applications. In particular, the measured values of around ∼1.02 and ∼1.78 eV of optical bandgaps were exhibited by phase-pure Cs_2_TiI_6_ and Cs_2_TiBr_6_, respectively; whereby the measurements were compared to a measured value of ∼1.51 eV for the optical bandgap of MAPbI_3_. These newly synthesized Ti-based double perovskites demonstrated stability and processability, thereby confirming the potential of their usage in PSCs. It was foreseen that further exploration of the Ti-based double perovskite family using the solution/vapor processing approach and material/device engineering would yield the benefits of having energy-saving, non-hazardous, inherently/ecologically stable, and low-cost perovskite solar cells [113].

Other sets of vacancy-ordered double perovskites are the tellurium-centered double perovskites. Ju and co-workers reported a family of Te-based lead-free perovskite A_2_TeX_6_ (A = MA, FA or BA; X = Br− or I−, MA = CH_3_NH_3_; FA = CH(NH_2_)_2_; BA = benzylamine) as potentially active materials for optoelectronic devices. These perovskites exhibited broad absorption (812–871 nm). Ideally, light-harvesting of up to 800 nm (the near-infra (NIR) region) of materials is preferable as light absorbers for potential applications in plain and simple solar cells, including NIR photodetectors. Moreover, the perovskite materials exhibited a tailoring bandgap (1.42–2.02 eV), a small trap density (∼1010 cm^−3^), and high mobility (∼65 cm^2^ V^−1^ s^−1^). MA_2_TeBr_6_, one of the members of the Te-centered double perovskite and with a bandgap of 2.00 eV, showed an encouraging result, with the aim of possessing a long carrier lifetime of ∼6 μs and a corresponding carrier diffusion length of ∼38 μm, which are ideal compounds for solar cells. Moreover, the perovskite compounds were found to be robust at ambient conditions, being stable for at least two months without showing any sign of phase change [114]. Hence, a summary of the ordered-vacancy double perovskites with their synthetic methods has been listed in Table 4.

## 4. Two-Dimensional (2D) Perovskite Absorbers

Two-dimensional (2D) perovskites, which are hybrid perovskites with low dimensionality, have received wide attention in the field of photovoltaics [115]. The two-dimensional (2D) perovskite absorbers are unique substances with molecular configuration structured in the form of (RNH_3_)_2_MX_4_, wherein R-NH_3_^+^conforms to different aliphatic or aromatic ammonium positively charged ions, X represents the halides, and M denotes various divalent metal ions. Their molecular structures are known to be body-centered tetragonal and comprise films of corner-sharing MX_6_ octahedra, whereby R-NH_3_ positively charged ions fill in the voids concerning the X negatively charged ions on either side of the films. These 2D perovskite structures present a great opportunity for flexibility when forming their structural materials and have been reported with different metallic ions, including Cu^2+^, Ni^2+^, Co^2+^, Fe^2+^, etc. with various ammonium cations [116]. As a result of the dimensionality reduction, the two dimensional (2D) perovskite absorbers show major changes concerning their optical properties due to strong quantum/dielectric confinement effects [117]. In terms of strong quantum wells, excitons gain more stability when compared to 3D perovskites due to the stronger coulomb transmission flanked by the electrons and holes in 2D perovskites [118]. The multi-quantum fine electronic structure in 2D perovskites presents particular magnetic and dielectric properties [116]. A typical 2D perovskite material has a value of up to 300 meV of exciton binding energy, and its self- built film exhibits photoluminescence at ambient temperature. One more important quality attribute of perovskite is the integrity of combing the organic flexibleness, inorganic movability, and toughness in a specific molecule range. This creates the opportunity for tuning their optical and electrical properties by way of varying either the organic or inorganic constituents [119].

Among many synthesized 2D perovskites obtained by a different method, there is a unique category known as Ruddlesdenepopper (RP) perovskites, which have similarities to those of conventional 2D perovskite materials in possession of a van der Waals layered crystal structure [120]. The Ruddlesdenepopper (RP) perovskites are represented by chemical formula (RNH_3_)_2_A_n−1_Pb_nx3n+1_, where R represents aromatic or aliphatic alkylammonium cation A^+^ is MA^+^ or FA^+^, X− stands for the halogen ion, and n represents the number of layers of perovskite films [121,122]. A significant difference to typical layered perovskites is that this special kind of perovskite has naturally integrated quantum well structures with quantum confinement effects without thinning of the atomic thickness. Hence, these sets of perovskites are also termed as 2D or quasi-2D perovskites [120]. The unique environmental stabilities of Ruddlesdenepopper (RP) perovskites, when exposed to moisture, can be attributed to the hydrophobicity of the larger organic cation, which prevents water molecules from attacking the inorganic layers. However, 2D-Ruddlesdenepopper (RP) perovskite solar cells have displayed poor efficiency (only 4–5%) [121]. Hence, the need to improve the power efficiency as well as replacement of toxic lead has led to the formation of potential 2D layered-Ruddlesdenepopper (RP) perovskites through the introduction of substituents of transition metals such, as copper, iron, palladium, zinc, and manganese. Herein, a summary of device performance on selected 2D lead-free halide perovskite solar cells is listed in Table 5.

### 4.1. Cu-Based 2D Perovskite Absorbers

**Figure 10:**
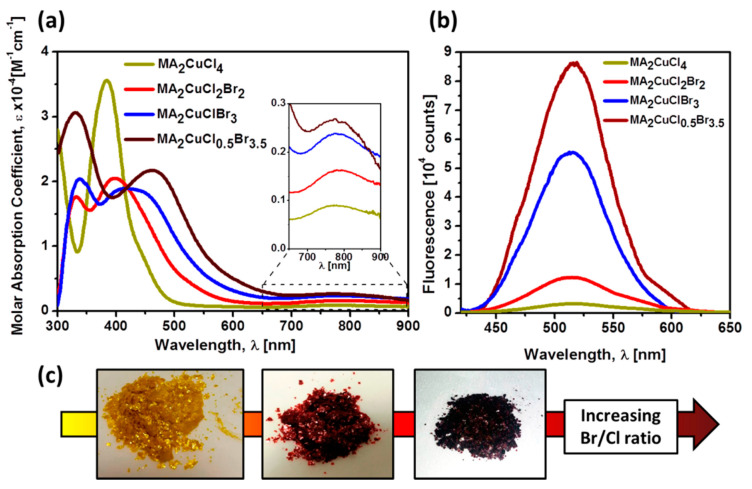
(**a**) Absorption coefficient series of MA_2_CuCl_x_Br_4−x_ by way of d−d charge transfer between 700 and 900 nm (**b**) Photoluminescence of MA_2_CuCl_x_Br_4−x_ (λexc = 310 nm); (**c**) color shift for powders: MA_2_CuCl_4_ (yellow), MA_2_CuCl_2_Br_2_ (red), MA_2_CuCl_0.5_Br_3.5_ (dark brown). Reproduced with permission [123]. Copyright: American Chemical Society (2016).

Cortechia and coworkers were the first to demonstrate and report the possible use of 2D copper perovskite through absorbers and provided a background intended for more studies on the development of transition metal-based perovskites as lead-free replacement materials. A sequence of (CH_3_NH_3_)_2_CuClxBr_4−x_, based on a starting material of an aliphatic amine, was studied, whereby the role of the Br/Cl ratio was found to be responsible for material stability and optical characteristics. The exploitation of added Cu d–d charge transfer and fittingly tailoring the proportion of Br/Cl, which likewise influences ligand-to-metal charge transfer transitions, extended the optical absorption in the sequence of the compound to near-infrared for optimum spectra-overlaying irradiance. In the sequence of (CH_3_NH_3_)_2_CuClxBr_4−x_, it was observed that their correlated values of 2.48 eV (500 nm) for MA_2_CuCl_4_ and 1.8 eV (689 nm) for M_A2_CuCl_0.5_Br_3.5_ optical bandgaps could be tailored by accruing the Br substance, whereas a further provision can be implemented for the absorption within the range of 700 and 900 nm on or after transitions within d Cu levels as shown in Figure 10. Besides, the charge transfers and d−d transitions were displayed in the direction that strongly results in photocurrent production. Using MA_2_CuCl_2_Br_2_ as a sensitizer of the series, a value of 0.0017% was obtained for power conversion efficiency [123].

Similarly, Elseman et al. reported a series of Copper-centered mixed perovskite materials, with the common formulation of (CH_3_NH_3_)_2_CuX (X = Cl_4_, Cl_2_I_2_, and Cl_2_Br_2_) for perovskite solar cells. The chlorine ion (Cl) in the structure was found to be responsible for the stabilization of the formed compound. The obtained results showed that (CH_3_NH_3_)_2_CuCl_4_ produced an optical bandgap of 2.36 eV and a power conversion efficiency of 2.41%, whereas they were 0.99 eV and 1.75% for (CH_3_NH_3_)_2_CuCl_2_I_2_ and 1.04 eV and 0.99% for (CH_3_NH_3_)_2_CuCl_2_Br_2_. It was observed that (CH_3_NH_3_)_2_CuCl_2_Br_2_ provided a far lesser conversion efficiency despite its optimized bandgap. The lower performance of (CH_3_NH_3_)_2_CuCl_2_Br_2_ can be explained due to Cu^2+^ reduction caused by the higher trap density. Green photoluminescence of the perovskite materials was achieved due to Cu^+^ ions [125]. However, Li and coworkers reported an aromatic amine of the 2D-layered (C_6_H_5_CH_2_NH_3_)_2_CuBr_4_ perovskite. The compound exhibited a bandgap value of 1.81 eV, whereby at an intense absorption of 539 nm, it gave rise to a high absorption coefficient with a value of around ∼1 × 10^5^ cm^−1^, thereby inferring its suitability for photon harvesting in thin-film solar cells. The analytical results of XRD, UV–vis absorption, and TGA confirmed the compound’s significant stableness when exposed to humidity, heat, and ultraviolet light. By perovskite exploitation in mesoscopic solar cells, a 0.2% conversion efficiency was obtained [126].

Meanwhile, Hajiaoui and co-workers synthesized a copper-based hybrid perovskite using chlorine substituted in an aliphatic amine as a starting precursor. A novel layered 2D perovskite material of [Cl(CH_2_)_2_-NH_3_]_2_[CuCl_4_] was developed, wherein 2-chloroethylammonium positively charged ions fills the voids surrounding the CuCl_6_ octahedra. The structural analysis indicates that the phase transition in the area of T1 = 281 K is induced by an unusual boat-to-chair conformation change of some of the 2-chloroethylammonium cations and the reorientation displacement of [CuCl_6_]_n_^4−^ zigzag chains. The perovskite material exhibited an indirect bandgap equal to 1.98 eV. The small activation energy (0.26 eV) at low temperature indicates that conduction, with the measurement between 10^−5^ and 10^−4^ Ω^−1^ m−1 in the material, can be assured of electronic conduction [127].

### 4.2. Fe-Based 2D Perovskite Absorbers

By exploring the significant structural diversity on compounds with the common molecular formula A_2_MX_4_, whereby A = organic positively charged ions, M = transition metal ions, X = halide negatively charged ions, it is well known that perovskite-related layered structures with octahedral-coordinated M atoms are found, for example, in (CH_3_NH_3_)_2_MCl_4_ (M = Cu, Mn, Cd, Fe). However, a great number of structures with central M atoms are tetrahedrally coordinated, for example, in (CH_3_NH_3_)_2_MCl_4_ (M = Zn, Hg) [128]. Even though structures with octahedral-coordinated M atoms are found in (CH_3_NH_3_)_2_FeCl_4_ in the family of A_2_MX_4_, Yin and coworkers were the first to report regular tetrahedron structures found in orthorhombic CH_3_NH_3_FeCl_4_, which is not in the family pattern of A_2_MX_4_; thus, they were used to investigate its structure, adsorption properties, and photoelectric behavior. Due to the presence of four Cl ligands coupled with a d^5^ of Fe^3+^ and FeCl^4−^ions, The CH_3_NH_3_FeCl_4_ compound was formed with a bandgap value of almost 2.15 eV. The observed values of the three-emission luminescence were 398, 432, and 664 nm, respectively. The solar device built on the assembly of the FTO/TiO_2_/MAFeCl_4_/carbon electrode attained photoelectric conversion efficiency of 0.054% with a value of 0.319 V voltage source, a photocurrent of 0.375 mA cm^−2^, and a 0.45 fill factor under an AM1.5, 100 mW cm^−2^ simulated illumination [124].

### 4.3. Pd-Based 2D Perovskite Absorbers

Even though the magnitude bounds projected through the Goldschmidt tolerance factor formula were higher than the magnitude of the organic cation of Pd-based 2D perovskites, Huang and co-workers showed the preparation of a previously unknown (CH_3_NH_3_)_2_PdCl_4_ compound. The prepared material has a compounds phase bulk resistivity value of 1.4 Ωcm^−1^, a direct bandgap of 2.22 eV, and an absorption coefficient of 10^4^ cm^−1^. The XRD analysis of (CH_3_NH_3_)_2_PdCl_4_ showed to be moderately stable in the air when compared to numerous present in hybrid perovskites that are disposed to phase deterioration when open to ambient air [18]. Furthermore, Zhou et al. synthesized a series of organic–inorganic layered Pd-centered perovskites, such as (CH_3_NH_3_)_2_PdCl_4_, (CH_3_NH_3_)_2_PdCl_4−x_Br_x_, and (CH_3_NH_3_)_2_Pdl_3_ and studied the adsorption properties and photoelectric behavior. The (CH_3_NH_3_)_2_PdCl_4_ compound exhibited an absorption band of 600 nm with a bandgap value of 2.15 eV, while (CH_3_NH_3_)_2_PdCl_4−x_Br_x_, displayed a band of 700 nm with a bandgap of 1.87 eV, as well as a band of 1000 nm coupled with a bandgap value of 1.25 eV for (CH_3_NH_3_)_2_PdCl_4−x_Br_x_. Interestingly, the photoelectric response of CH_3_NH_3_PdI_3_ reached 950 nm. The results have drawn attention in the fields of optoelectronics and photovoltaics [129].

### 4.4. Mn-Based 2D Perovskite Absorbers

By exploiting the impressive luminescent and photoelectric properties, 2D layered Pb-free hybrid perovskites have exhibited practical applications in optoelectronic and photovoltaic devices [130]. Nie et al. reported the photoresponse of (CH_3_NH_3_)_2_MnCl_4_ in a photoelectric device. The solution-processed (CH_3_NH_3_)_2_MnCl_4_ thin layer displayed its position alongside the b-axis route on the TiO_2_ area. The photoelectric cell through the FTO/TiO_2_/(CH_3_NH_3_)_2_MnCl_4_/ carbon electrode showed evident photoresponses detected beneath 10−30 Hz flashlight frequencies and a 330 nm light beam. This modest photoresponsive device may be impactful in the future for industrialized assembly of photosensitive recorders and memory devices [130].

Furthermore, Cheng et al. reported centimeter-size square 2D-coated single crystals of (CH_3_NH_3_)_2_MnCl_4_ perovskite. The single crystals of (CH_3_NH_3_)_2_MnCl_4_ were developed by a varied crystal network (squares and octagon) using different organic solvents and concentrations of hydrochloric acid solution. Comparing the powder XRD analysis of both (CH_3_NH_3_)_2_MnCl_4_ single crystals, the results showed that they equally fit into the equivalent crystal coordination and have equivalent cell factors. Photons can sensitize these materials in diverse wavelength bands. The key band remains situated at 608 bases on its emission spectra and is placed at 72 nm full width at half maximum (FWHM) through an excellent elongated lifetime of microseconds.

Moreover, LED appliances’ optoelectronic application was organized based on the (CH_3_NH_3_)_2_MnCl_4_ single crystals, with LED being prepared for the single crystals of (CH_3_NH_3_)_2_MnCl_4_. The results showed that the single crystal of (CH_3_NH_3_)_2_MnCl_4_ has suitable luminescence effects, can radiate evident red light, and be applied for white light illumination. Hence, the unique luminescence properties of the (CH_3_NH_3_)_2_MnCl_4_ single crystal equally show that it can be used for the application of perovskite solar cells [24].

## 5. Perovskite-Like Halide Absorbers (A_3_B_2_X_9_)

The compound halides of bismuth and antimony all possessing a valence of three are known to not have the ability to adapt the same perovskite structures of three-dimensional (3D) perovskite frameworks with common formula of ABX_3_. These compositions are inclined to develop zero-dimensional, one-dimensional, and two-dimensional (2D) perovskite-like halide absorbers with properties featuring varying sizes agreeing to the direction of measurement due to the purpose of quantitative relations between the reactants [27]. These perovskite-like halide absorbers are a structurally rich group of mixed organic−inorganic halide perovskites from the extensive family of A_3_B_2_X_9_ structures, where A represents monovalent positively charge ions = Cs^+^, Rb^+^, or CH_3_NH_3_^+^ alias MA^+^; B stands for the positively charged trivalent metal ions = Bi^3+^, Sb^3+^; and X denotes negatively charged ions of halides = (Cl^−^, Br^−^, or I^−^) employed as active photovoltaic absorbers [45,131,132,133].

**Figure 11:**
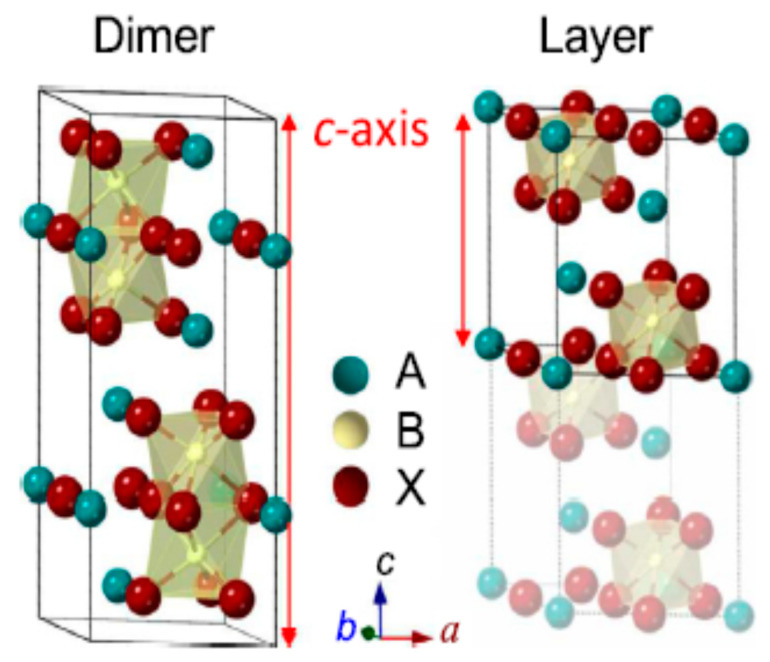
The schemes showing crystal structures of dimer- and layer-type A_3_B_2_I_9_. Reproduced with permission [133]. Copyright: Elsevier Ltd. American (2019).

In the group of A_3_B_2_X_9_ compositions, the A and X atoms are situated in the orientation of closest packing, and B atoms tend to fill two-thirds of the octahedral X_6_ voids. The two main types of A_3_B_2_X_9_ compounds are the 0-D dimer phase of hexagonal close packing and the 2-D layered phase of cubic tight packing of A and X atoms [134], as shown in Figure 11. When small cations (Cs or Rb) are used, the two-dimensional (2D) layered perovskite-like structures are formed. Meanwhile, the zero-dimensional (0D) dimers are produced when large cations (e.g., MA) are used. The Sb- and Bi-based perovskites are the two kinds of materials that are more stable in air and moisture than Pb-based perovskites [135]. Herein, a summary of device performance on selected lead-free perovskite-like solar cells is displayed in Table 6.

### 5.1. Sb-Based Perovskite-Like Halides

Antimony is a group 15 element, and existing in its stable + 3 states can form either a 2-D layered perovskite structure or a 0-D dimer [135]. Generally, when a trivalent metal positively charged ion of Sb^3+^ replaces a bivalent heavy metal positively charged ion of (Pb^2+^) in ABX_3_ perovskite, it results in the transformation of a defect-order structure of A_3_B_2_X_9_ perovskite [136]. These antimony-based perovskite-like halide materials belong to the family of A_3_B_2_X_9_ perovskites. Saparov and coworkers were the first to report the 2D composition of the layered pattern of Cs_3_Sb_2_I_9_ that has a bandgap value of 2.05 eV, whereby this experimental value is in alignment with the calculated theoretical value of 2.06 eV [146]. Furthermore, Singh et al. reported the inorganic layered Sb-based perovskite Cs_3_Sb_2_I_9_ using solution processing to obtain an optical bandgap value of 2.05 eV. A solar cell device with an assembly of inorganic layered Cs_3_Sb_2_I_9_ perovskite (ITO/PEDOT (PSS poly(3,4-ethylenedioxythiophene) poly(styrenesulfonate)): PSS/Cs_3_Sb_2_I_9_/PC_71_BM/Al) displayed a value of 0.72V for its voltage source, a photocurrent of 5.31 mA cm^−2^, and a value of 0.39 for its fill factor, thereby reaching power conversion efficiency of 1.49% under AM1.5G solar illumination. Using the principal device to integrate the dimer model displayed a voltage source value of 0.77 V, a photocurrent of 2.82 mA cm^−2^, and a fill factor of 0.40, thereby reaching a PCE of 0.89% [137].

Through the solvent engineering method with a toluene drop throughout the spin-coating procedure, Hebig et al. introduced the methylammonium antimony iodide (CH_3_NH_3_)_3_Sb_2_I_9_ 0D perovskite as a promising material with the aim of producing lead-free solar devices. The unstructured layers of (CH_3_NH_3_)_3_Sb_2_I_9_ were determined to have a peak absorption coefficient of around α ≈ 10^5^ cm^−1^ and optical bandgap of 2.14 eV. A planar heterojunction device was fabricated to ascertain the potential of (CH_3_NH_3_)_3_Sb_2_I_9_ as a light harvester for photovoltaic applications, which yielded around η ≈ 0.5% conversion efficiency, previously presenting a defined fill factor of 55% and a voltage source of 0.89 V, but low photocurrent densities. Through enhancing the contact layers and the morphology of the Sb-perovskite, far greater power conversion efficiencies may be achievable [138].

Recently, Karuppuswamy et al. (2018) employed antisolvent conduct to the surface morphology enhancement of the Sb-centered dimer of (CH_3_NH_3_)_3_Sb_2_I_9_ crystals by speeding up heterogeneous nucleation. This process was achieved by incorporating an interlayer that performed as favorable hydrophobic support for the growth of large-grain (CH_3_NH_3_)_3_Sb_2_I_9_ crystals, thereby reducing the number of spaces and growing the film property. A bandgap of 1.9 eV was obtained. By incorporating the Sb-based perovskite-like photoabsorbers as the active layer on fabricated inverted planar heterojunction PSCs, the photovoltaic properties displayed a voltage source value of 0.77 V resulting from a photocurrent of 6.64 mA cm^−2^ and a fill factor of 59.60%, thereby reaching a PCE of 2.77% [135].

By replacing the A cation of either Cs or CH_3_NH_3_ with Rb cation, Harikesh and co-workers demonstrated the solution-processed Sb-based perovskite of Rb_3_Sb_2_I_9_, which obtained values of 2.24 and 2.1 eV of direct and indirect bandgaps. The fabricated solar device exhibited a voltage source of 0.55 V and a photocurrent of 2.12 mA/cm^2^ short circuit current density, and it achieved efficiencies of up to 0.66% [139]. Recently, Correa-Baena et al. reported Sb-based compounds of 2D layered Rb_3_Sb_2_I_9_ which achieved a 2.03 eV direct bandgap and yielded a 0.76% power conversion efficiency, including features such as a voltage source of 0.66 V, a photocurrent of 1.84 mA cm^−2^ and a fill factor value of 0.63 [147]. Furthermore, Weber et al. investigated the control of changing the bromide proportion to the iodide proportion on the structural, optical, and photovoltaic properties of Rb_3_Sb_2_Br_9−x_ I_x_ (x = 0–9). Sequential replacement of iodide with the lesser bromide does not alter the crystal coordination; however, compounding the bromide substance ends in a reduction of the unit cell, in addition to in a blue shift of the absorption onset, raising the bandgap from 2.02 to 2.46 eV. The fabricated unveiled solar cells with Rb_3_Sb_2_I_9_ (Px = 0–9), as the light harvester exhibited photovoltaic properties presenting values of Voc = 0.55 V, Jsc = 4.25 mA cm^−2^, and fill factor = 59.5%, reaching a PCE of 1.37% [140].

Based on replacing the A cations of either Cs or CH_3_NH_3_ with NH_4_ and adjusting the halide, Zuo et al. prepared an (NH_4_)_3_Sb_2_I_x_Br_9−x_ (0 ≤ X≤ 9) group of perovskite absorbers by using an anti-solvent vapor-assisted crystallization method. The light-harvesting of the (NH_4_)_3_Sb_2_I_x_Br_9−x_ perovskite material was tailored by modifying I and Br content. The family members such as (NH_4_)_3_Sb_2_I_9_, (NH_4_)_3_Sb_2_I_6_Br_3_, (NH_4_)_3_Sb_2_I_3_Br_6_, and (NH4)_3_Sb_2_Br_9_ layers show direct optical bandgaps of 2.27 eV,2.49 eV, 2.66 eV and 2.78 eV, respectively. The atomic force microscope (AFM) analyzed the (NH_4_)_3_Sb_2_I_9_ layer, and the result showed that the layer had somewhat compact and uniform morphology. Upon using the formation of ITO/PEDOT:PSS/(NH_4_)_3_Sb_2_I_9_/PC_61_BM/Al in solar cells, the performing photovoltaic characteristics of (NH_4_)_3_Sb_2_I_9_ by way of the end member were investigated to give a Voc of 1.03 V, a Jsc of 1.15 mAcm^−2^, a fill factor of 42.88%, and a PCE of 0.51%. The Voc of the (NH_4_)_3_Sb_2_I_9_ perovskite solar cell is known to be much higher than the Voc of most lead-free perovskite solar cells. Hence, the improvement of (NH_4_)_3_Sb_2_I_9_ perovskite crystallinity will increase hole and electron mobilities and enhance the power conversion efficiency [141].

**Figure 12:**
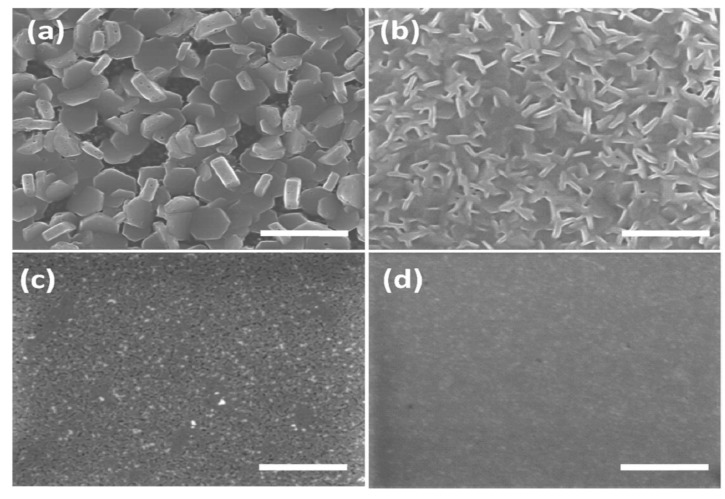
SEM images of MA_3_Sb_2_I_9_ (**a**) without HI and (**b**) with HI, and Cs_3_Sb_2_I_9_ (**c**) without HI and (**d**) with HI. Perovskites prepared on an ITO/ PEDOT: PSS substrate at optimized molar ratios and additive concentrations; scale bar 2 mm. Reproduced with permission [142]. Copyright: The Royal Society of Chemistry (2017).

Comparing the performance of Sb-based perovskite-like halides, Boopathi and co-workers demonstrated a single-step technique to formulate solution-processable (CH_3_NH_3_)_3_Sb_2_I_9_ and Cs_3_Sb_2_I_9_ perovskite thin films. The use of precursor molar ratios and HI additive concentrations produced stoichiometric perovskite films, whose crystalline phases were analyzed through XRD. The new HI addictive technique gave rise to greatly improved perovskite thin film. SEM images of processable (CH_3_NH_3_)_3_Sb_2_I_9_ and Cs_3_Sb_2_I_9_ perovskite materials exhibited different morphologies due to their differences in light absorption and device performance. The optical bandgaps of (CH_3_NH_3_)_3_Sb_2_I_9_ and Cs_3_Sb_2_I_9_ were evaluated as 1.95 and 2.0 eV and were influenced by their respective morphologies, as shown in Figure 12. Using (CH_3_NH_3_)_3_Sb_2_I_9_ perovskite material in an assembly of planar device architecture solar cells, a PCE of 2.04% was obtained. However, for the assembly of the solar devices with CH_3_NH_3_)_3_Sb_2_I_9_ perovskite material, a PCE of 0.5% was previously obtained by Hebig and co-workers as well as Zuo and co-workers, which remained lower than the present value of 2.04%. This is in the right step towards achieving perovskite-like halides to serve as materials for photovoltaic application. The photovoltaic performance parameters of champion Sb-based perovskite devices fabricated with and without the HI additive such as with bare MA_3_Sb_2_I_9_ yielded a PCE of 1.11% and a voltage source of 0.6 V; MA_3_Sb_2_I_9_ + HI obtained a 2.04% PCE and a V_OC_ of 0.62 V; bare Cs_3_Sb_2_I_9_ attained a 0.67% PCE and a voltage source of 0.62 V; and Cs_3_Sb_2_I_9_ + HI yielded a PCE of 0.84% and a voltage source of 0.60 V [142].

To resolve the subject of the wide optical bandgap linked by the (CH_3_NH_3_)_3_Sb_2_I_9_ perovskite compound, Chatterjee and Pal introduced Sn^4+^ at the metallic position of the antimony-based defect-ordered mixed iodide (CH_3_NH_3_)_3_Sb_2_I_9_ perovskite structure, resulting in a steady shift in the electronic transfer by the perovskites with an optical gap value of 2 eV which was successfully lowered to around 1.55 eV. Using the scanning tunneling spectrometry and density-of-state spectra analysis, a remarkable shift of Fermi energy in the direction of the conduction band edge occurred due to a rise in the Sn substance held within the perovskite. This shift brought about in tailoring a kind of electronic potential from the p-type to the n-type and, essentially, led to a better bandgap configuration with the selective connections of p–i–n heterojunctions. Nevertheless, the surface coarseness of the perovskite layer was adversely affected due to tin inclusion. Therefore, the tin substance was enhanced by taking prevailing factors, namely, the bandgap of the compound and the surface coarseness of thin layers, into account. The fabricate heterojunction device with 40% tin as a substitute for antimony enshrined in (CH_3_NH_3_)_3_(Sb_1−x_ Sn_x_)_2_I_9_ perovskite exhibited the following photovoltaic properties: a voltage source value of 0.56 V, a photocurrent of 8.32 mA cm^−2^ and a fill factor of 58%, reaching a PCE of 2.69% [136].

Moreover, in a bid to resolve the issue of deprived layer morphology and overwhelming halide components associated with the (CH_3_NH_3_)_3_Sb_2_I_9_ perovskite material, which are results of the disorder of growth progression, Yang et al. introduced bis(trifluoromethane)sulfonimide lithium (LiTFSI) into (CH_3_NH_3_)_3_Sb_2_I_9_ perovskite material to produce high-level property two-dimensional (CH_3_NH_3_)_3_Sb_2_I_9−x_Cl_x_ layers. Through the linker molecule surrounded by Sb-based pyramidal groups, LiTFSI is responsible for providing a zero-dimensional in-between state and impeding crystallization. The gradual conversion of dimensions will steady the bandgap of perovskite-like layers with a constant Cl/I ratio (∼7:2), preventing the arbitrary “x” quantity in (CH_3_NH_3_)_3_Sb_2_I_9−x_Cl_x_ layers made from the traditional technique. By using this technique, Sb-based perovskite-like solar cells (PLSCs) obtained a maximum power conversion efficiency (PCE) of 3.34% and retained 90% of the preliminary PCE when kept under ambient surroundings for about 1400 h [143].

### 5.2. Bi-Based Perovskite-Like Halides

**Figure 13:**
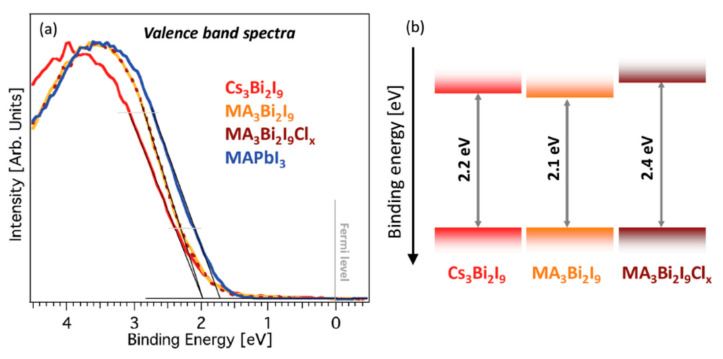
(**a**) Valence level spectra of the Cs_3_Bi_2_I_9_, MA_3_Bi_2_I_9_, and MA_3_Bi_2_I_9_Cl_x_ samples measured by XPS using AlK α in red, orange, and brown (dotted line), respectively. As a comparison, a valence level spectrum of MAPbI_3_ recorded in the same conditions is presented in blue. The intensities at the valence band peaks are set to the same intensity for simple comparison. (**b**) Schematic drawing of the energy level diagram versus the Fermi level. Reproduced with permission [151]. Copyright: WILEY-VCH Verlag GmbH & Co. KGaA, Weinheim (2015).

Bismuth belongs to a group of 15 elements and is the only one among the 6p block elements with an outer lone pair of 6s^2^ electrons of lead [148]. Due to the Bi^3+^ outer lone pair of 6s^2^ electrons, the equal numbers of electrons or equivalent electronic structure, and the way by which its electron cloud is deformed by electric fields, as seen in Pb^2+^, bismuth-based perovskite-like halide crystal chemistry is related to the lead halide perovskite such that the rich structural diversity of the Bi-based compound is detected containing deformation, space sites, and numerous methods for the combination of the MX_6_ octahedra. However, Bi^3+^ has a propensity to form compositions of subordinate dimensionality when compared to the metal halide groups of Pb^2+^ [134]. The formed structures of lower dimensionality vary from systems based on isolated (0D) inorganic polyhedral to one-dimensional (1D) ones with extended chains, right up to two dimensional (2D) networks [149]. The first crystal structure of bismuth perovskite of Cs_3_Bi_2_I_9_ was studied in the 1960s. After a period of 50 years, Park and co-workers were the first to incorporate bismuth perovskite of Cs_3_Bi_2_I_9_ into solar cells [150]. Using a one-step spin coating method, Park et al. prepared the perovskite materials of Cs_3_Bi_2_I_9_, MA_3_Bi_2_I_9_, and MA_3_Bi_2_I_9−x_Clx. XRD analysis showed that all samples were of a hexagonal crystalline phase and in the space group P6_3_/mm. The three materials, MA_3_Bi_2_I_9_, MA_3_Bi_2_I_9_Cl_x_, and Cs_3_Bi_2_I_9_, were analyzed through an X-ray photoelectron spectroscopy, whereby the estimated I/Bi ratios, such as 4:4, 4:7, and 4:6, were used for their formation. Hence, the investigation gives rise to the valance band spectra and energy level diagram shown in Figure 13. The bandgaps were approximated to be around 2.1, 2.2, and 2.4 eV for the MA_3_Bi_2_I_9_, Cs_3_Bi_2_I_9_, and MA_3_Bi_2_I_9−x_Cl_x_ samples. Among the perovskites, Cs_3_Bi_2_I_9_ recorded the best photovoltaic parameters, which were determined as a photocurrent of 2.15 mAcm^−2^, a voltage source of 0.85 V, and a fill factor value of 0.60, and they attained a PCE of 1.09%. Hence, the recorded power conversion of the Cs_3_Bi_2_I_9_ perovskite material was greater than the two other perovskites: a PCE of η = 0.12% for MA_3_Bi_2_I_9_ and η = 0.003% for MA_3_Bi_2_I_9−x_Cl_x_ [151].

Recently, Ma and co-workers also reported also the synthesis of millimeter-scale single crystals of Cs_3_Bi_2_I_9_ and MA_3_Bi_2_I_9_ perovskites via a facile hydrothermal approach. The Cs_3_Bi_2_I_9_ and MA_3_Bi_2_I_9_ single crystals exhibited similar light absorption properties, both holding an approximated bandgap of 1.9 eV. Spin-coating of these compounds produced thin films with uniform surface morphologies, superior carrier mobility, and more stability. By assembling a solar cell (utilizing a spin-coating procedure), the optoelectronic properties of these compounds were tested. The solar device with the MA_3_Bi_2_I_9_ compound obtained a 0.2% PCE with the following parameters: a voltage source of 0.53 V, a photocurrent of 0.65 mA cm^−2^, and a fill factor value of 0.57. Similarly, the Cs_3_Bi_2_I_9_ perovskite solar cell obtained a PCE of 0.18%, which was achieved with Jsc = 0.58 mA cm^−2^, Voc = 0.54 V, and FF = 57%. The power conversion efficiencies obtained in these studies were higher than the PCEs reported by Park et al. (2015). These findings indicate that the electrical properties of these compounds could further be optimized to enhance the performance of photoelectric devices [152].

By XRD analysis, CsBi_3_I_10_ thin film has a layered composition with varying control of crystal development when compared to the Cs_3_Bi_2_I_9_ perovskite. A bandgap value of 1.77 eV was achieved for of CsBi_3_I_10_ film, while a bandgap value of 2.03 eV was achieved for of Cs_3_Bi_2_I_9_ perovskite.

Johansson and coworkers reported the photo-conversion of non-toxic bismuth materials, such as Cs_3_Bi_2_I_9_ and CsBi_3_I_10_, and a comparison of their morphologies was made by Park et al. in 2015 with a focus on Cs_3_Bi_2_I_9_ perovskite material [153]. By XRD analysis, CsBi_3_I_10_ thin film has a layered composition with varying control of crystal development when compared to Cs_3_Bi_2_I_9_ perovskite. A bandgap value of 1.77 eV was achieved for CsBi_3_I_10_ film, while a bandgap value of 2.03 eV was achieved for Cs_3_Bi_2_I_9_ perovskite. The light absorption of CsBi_3_I_10_ showed a band of up to 700 nm, while Cs_3_Bi_2_I_9_ perovskite showed a band of up to 600 nm. The improved light absorption based on CsBi_3_I_10_ when compared to that of Cs_3_Bi_2_I_9_ perovskites was attributed to its surface morphology having uniform coverage [153]. Recently, Ghosh et al. 2018 critically assessed Cs_3_Bi_2_I_9_ to have an approximated bandgap of 2 eV and noted it as a composition designed for a thin layer light harvester with a conversion efficiency that was described as firmly deficient due to the low photocurrent density. Therefore, Ghosh et al. 2018 suggested that by varying the stoichiometry of the starting materials, the power conversion efficiency of the Cs_3_Bi_2_I_9_ solar cell would be somewhat enhanced [154].

To corroborate and improve on the work of Park et al. in regard to the MA_3_Bi_2_I_9_ perovskite compound, other scientists reported the synthesis of the MA_3_Bi_2_I_9_ perovskite compound featuring different photovoltaic properties. Oz et al. reported that the MA_3_Bi_2_I_9_ perovskite compound exhibited a wide bandgap value of 2.9 eV, and, upon optical excitation, photoluminescence emission was recorded at 1.65 eV (751 nm). The PCE was close to 0.1% upon the fabrication of MA_3_Bi_2_I_9_ layers in a solar cell with a planar heterojunction configuration (ITO/PEDOT/ (CH_3_NH_3_)_3_Bi_2_I_9_/PCBM/Ca/Al) [155]. Singh et al. reported the slightly improved photovoltaic performance with 0.2% PCE through an appropriate cell configuration of MA_3_Bi_2_I_9_ composition using planar, brookite, and anatase mesoporous layers for a structured photovoltaic perovskite [131]. Zhang et al. reported the enhanced efficiency of MA_3_Bi_2_I_9_-based solar cells, reaching 0.42% with mesoscopic architecture on a TiO_2_/ITO substrate through a one-step spin-coating. Relatively high values were exhibited: Voc = 0.66 V and fill factor = 62.48% [156].

Furthermore, Ran et al. reported a plane, constant, and dense MA_3_Bi_2_I_9_ thin film being produced through a new two-step evaporation–spin-coating layer invention approach [157]. Using an inverted planar heterojunction photovoltaic device, the MA_3_Bi_2_I_9_ thin film exhibited a value of Voc = 0.83 V, reaching a PCE of 0.39% [157]. Zhang et al. reported the adoption of a two-stage technique—high-vacuum BiI_3_ discharge and low-vacuum uniform MA_3_Bi_2_I_9_ conversion—to harvest dense, pinhole-free, and fine-crystallized MA_3_Bi_2_I_9_ layers with large submicron-micron granules. Due to the ideal morphologies of MA_3_Bi_2_I_9_ when compared to previously synthesized MA_3_Bi_2_I_9_ perovskites, the target MA_3_Bi_2_I_9_ films in the solar cell device exhibited a 1.64% PCE with all three J-V parameters displaying a voltage source value of 0.83 V, a short circuit current of 3.00 mA cm^−2^, and a fill factor value of = 0.79, overcoming or, in the same way, reaching the best values to date for MA_3_Bi_2_I_9_ solar cells [144]. However, Jain et al. reported the enhancement in crystallinity and surface morphology of methylammonium bismuth iodide (MAI), whereby through a controlled, stepwise formation of methylammonium bismuth iodide (CH_3_NH_3_)_3_Bi_2_I_9_ perovskite films synthesized via the vapor-assisted solution process (VASP) by exposing BiI_3_ films to CH_3_NH_3_I (MAI) vapors for several reaction times, (CH_3_NH_3_)_3_Bi_2_I_9_ semiconductor layers with tailoring optoelectronic characteristics were attained. With good reproducibility, solar cells prepared on mesoporous TiO_2_ substrates obtained hysteresis-free efficiencies of up to 3.17%. This good performance is attributed mainly to the uniform surface coverage, enhanced stoichiometry, lowered metallic substance in the bulk, and the anticipated optoelectronic attributes of the absorbers [158].

Through A-site cation substitutions of either Cs or CH_3_NH_3_/MA with NH_4_ cation, Sun et al. reported the synthesis of (NH_4_)_3_Bi_2_I_9_ perovskite, which was made from the solution and composition elucidated by single-crystal X-ray diffraction. An approximated bandgap of 2.04 eV was obtained and, thus, was considered to be lower than that of CH_3_NH_3_PbBr_3_ at 2.20 eV [159]. Recently, Lan et al. in 2019 reported that the A-site cation substitutions in lead–halide perovskites altered their optical properties, and they therefore prepared a novel formamidinium (FA)-based bismuth perovskite material, (FA)_3_Bi_2_I_9_. (FA)_3_Bi_2_I_9_ showed a hexagonal phase with a more distended unit cell when compared with the traditional methylamine-based bismuth perovskite (MA)_3_Bi_2_I_9_. The perovskite compound exhibited a bandgap of 2.19 eV. The mesoporous-coordinated (FA)_3_Bi_2_I_9_ solar cells were assembled and showed a value of Voc = 0.48 V, reaching a PCE of 0.022%. Thus, they were considered to be a development allowing researchers to move beyond the methylamine-based bismuth perovskite solar cells, proposing a possible light harvester for lead-free perovskite solar cells [145]. Furthermore, Li et al. reported the synthesis of three novel organic–inorganic iodobismuthate containing organic positively charged ions with heterocyclic 5-membered rings by way of various inorganic structures beginning with 0D for [TH]_3_[Bi_2_I_9_] and [IM]_3_[Bi_2_I_9_], as well as ending with 1D [AT][BiI_4_] (TH = thiazolium, IM = imidazolium and AT = amino thiazolium). The direct optical bandgap values were redshifted from 2.08 eV for [TH]_3_[Bi_2_I_9_] and 2.00 eV for [IM]_3_[Bi_2_I_9_] to 1.78 eV for [AT][BiI_4_], determined by UV–vis reflectance spectroscopy. For electron injection purposes and to enable an enhanced energy level to match TiO_2_, the conduction band minimum (CBM) value of [AT][BiI_4_] was shifted to a lower value. Though the main influences of Bi 6p and I 5p antibonding associations were found for [AT][BiI_4_], theoretical studies showed that additional contribution of organic entities in the conduction band minimum could be found for [TH]_3_[Bi_2_I_9_]. By using [AT][BiI_4_] as the light absorber in a hole-conductor-free, completely printable solar cell with relatively good reproducibility, a power conversion efficiency of 0.47% was obtained [160].

## 6. Conclusions and Prospects

This review focused on the current understanding as to the substitution of lead via both homo- and hetero-valent substitution and its influence on various halide perovskite materials as well as their photovoltaic properties. Furthermore, the collective outcomes from the experimental findings on the hybrid lead-free halide metal perovskite were discussed with emphasis on how chemical compositions influence optical and morphological properties, and the limitations to aid future studies were identified.

Perovskite solar cells have inherently faced the drawback of upscaling the technology to the commercialization of energy and wide deployment of its outdoor applications due to the presence of harmful chemicals such as lead(II) halide materials in lead-based perovskites. The utilization of lead(II) halide materials in perovskite solar cells has raised environmental concerns, and a considerable number of questions has drawn attention to the feasibility of using solar cells technology that incorporates soluble lead(II) compounds due to toxicity, as well as the potential threat that the widespread deployment of the technology could pose on the environment. Moreover, these perovskite materials have questionable long-term stability. Hence, the metal substitution of lead in the perovskite composition can be essential by way of compositional fixing to tailor its optical, morphological, and electronic properties.

The structural dimensionality and the type of B cation to substitute for Pb are the two important factors that will determine the suitability of halide perovskite material for photovoltaic applications. This structural dimensionality and the type of B cations were reported in the following order of dimensions: 3D single perovskites, double perovskites, two-dimensional (2D), and perovskite-like halides with the common formula of A_3_B_2_X_9_. For the 3D perovskite, we observed the incomplete replacement of monovalent methylammonium (MA) positively charged ions by hydrazinium (HA) ions to enhance the stableness of MASnI_3_ films and improve their morphology, thereby giving rise to remarkable enhancement in the PCE of the solar cells. As regards the B positively charged ions, the replacement of Pb by Ge, Sn, Cu, Fe, Pd, Mn, Sb, or Bi obtained lower PCE values when compared to those described with MAPbI_3_.

The B cation is primarily the cation that governs the kind of perovskite crystal formation as well as the photovoltaic characteristics of the perovskite materials due to the specific electronic composition of each B cation. Moreover, for every given perovskite structure with the general formula of ABX_3_, their different chemical compositions of all the perovskite materials strongly determined their bandgaps. This is because the chemical composition made of different elements has different orbital energies and characters. The A cation is highly ionic and makes little contribution to the band edges. Therefore, the optical bandgap is mainly governed by the B cation and the X anion, which form the [BX_6_] octahedra framework. Furthermore, the optimization of the morphology is realized through mixed compositions. In some cases, morphology optimization is influenced through the concentrations of the perovskite precursors in the solution and the speed rotation.

Since the composition of the perovskite crystals directly controls the photovoltaic properties of the perovskite solar cells, the recommendation, therefore, is for systematic structural variations of unique components to be continuously carried out under the required reaction conditions in order to optimize their morphology properties via additives that suppress recombination due to the need for the exact amounts of components for hybrid perovskite.

## Figures and Tables

**Table 1:** Summary of device performance on selected 3D lead-free hybrid perovskite solar cells.

Lead-Free Halide Perovskite	E_g_ (eV)	Voc (V)	Jsc (mAcm^−2^)	FF	PCE (%)	Ref.
FA_0.75_MA_0.25_SnI_3_: SF_2_	1.33	0.61	21.2	0.63	8.12	[37]
MASnI_3_	1.3	0.68	16.30	0.48	5.23	[54]
MASnI_3−x_Br_x_	1.75	0.82	12.30	0.57	5.73	[54]
MASnIBr_0.8_Cl_0.2_	1.25	0.38	14.0	0.57	3.1	[55]
MA_0.8_HA_0.2_SnI_3_	-	0.38	14.1	0.47	2.6	[56]
FASnI_3_:SF_2_	1.41	0.238	24.45	0.36	2.10	[57]
FASnI_3_:10% en	1.51	0.48	22.54	0.66	7.14	[58]
FASnI_3_:N_2_H_5_Cl	1.37	0.455	17.64	0.67	5.4	[59]
Cs_0.08_FA_0.92_SnI_3_	-	0.44	20.70	0.67	6.08	[60]
(3D)FASnI_3_:(2D)Sn:SF_2_	-	0.525	24.1	0.71	9.0	[61]
20% SF_2_-CsSnI_3_	-	0.24	22.70	0.37	2.02	[62]
CsSnI_2.9_Br_0.1_	-	0.22	24.16	0.33	1.76	[63]
CsSnI_3_:Co(C_2_H_5_)	-	0.36	18.32	0.46	3.0	[64]
20% SF_2_-CsSnBr_3_	1.75	0.41	9.0	0.58	2.1	[65]
CsGeI_3_	1.63	0.074	5,7	0.27	0.11	[66]
MAGeI_3_	2.0	0.15	4.0	0.30	0.20	[66]
MAGeI_2.7_Br_0.3_	-	0.46	3.11	0.48	0.57	[67]

**Table 2:** Summary of device performance on selected metal halide double perovskite solar cells.

Metal Double Halide Perovskite	E_g_ (eV)	Voc (V)	Jsc (mAcm^−2^)	FF	PCE (%)	Ref.
Cs_2_AgBiBr_6_	1.91	1.01	3.19	0.66	2.2	[81]
Cs_2_NaBiI_6_	1.66	0.47	1.99	0.44	0.42	[82]
Cs_2_SnI_4_Br_2_	1.40	0.563	6.225	0.58	2.025	[83]

**Table 3:** Summary of metal halide double perovskites [84].

Material Compositions	Morphology	Bandgap (eV)	Synthetic Method	References
Cs_2_BiAgCl_6_	Crystal	2.2	Conventional solid-state reaction	[85]
Cs_2_Ag(Sb_x_Bi_1−x_)Br_6_	Smaller grains of mixed alloys	2.08	Solution-based route	[86]
Cs_2_AgBiBr_6_	Single crystal	1.72	Crystal engineering strategy	[87]
Cs_2_AgSbBr_6_	Single crystal	1.64	Hydrothermal methods	[88]
Cs_2_NaVCl_6_	Red crystals	2.64	Solid-state reaction and hydrothermal method	[89]
Cs_2_AgInCl_6_	Nanocrystals	3.57	Colloidal synthesis	[90]
Cs_2_AgSbCl_6_	Nanocrystals	2.57	Colloidal synthesis	[90]
Cs_2_CuSbCl_6_	Nanocrystals.	1.66	Modified one-pot hot injection of colloidal synthesis	[91]
Cs_2_NaBiI_6_	Single crystal	1.5	Solution-based method	[92]
(MA)_2_Au_2_X_6_, (X = Br, I)	Tetragonal crystal	1.0	Solution-processed route	[93]

**Table 4:** Summary of Ordered-vacancy double perovskites.

Material Compositions	Morphology	Bandgap (eV)	Synthetic Method	References
Cs_2_SnI_6_	Powders	1.84	Facile hydrothermal method	[105]
Cs_2_SnBr_6_	Powders	1.42	Facile hydrothermal method	[105]
Cs_2_SnCl_6_	Single-phase structures	4.89	Solution processing method	[106]
Cs_2_SnBr_6_	Single-phase structures	3.23	Solution processing method	[106]
Cs_2_SnI_6_	Single-phase structures	1.35	Solution processing method	[106]
Cs_2_PtI_6_	Cubic crystal	1.4	Solution processing method	[107]
Cs_2_TiBr_6_	Crystalline equiaxed grains	1.8	Two-step vapour deposition method	[108]

**Table 5:** Summary of device performance on selected 2D lead-free halide perovskite solar cells.

2D Lead-Free Halide Perovskite	E_g_ (eV)	Voc (V)	Jsc (mAcm^−2^)	FF	PCE (%)	Ref.
MACuCl_0.5_Br_3.5_	1.8	0.29	21 × 10^−6^	0.28	0.017	[123]
MAFeCl_4_	2.15	0.319	0.375	0.45	0.054	[124]

**Table 6:** Summary of device performance on selected lead-free halide perovskite-like solar cells.

Lead-Free Halide Perovskite-Like Absorbers	E_g_ (eV)	Voc (V)	Jsc (mAcm^−2^)	FF	PCE (%)	Ref.
(MA)_3_Sb_2_I_9_:antisolvent treatment	1.9	0.77	6.64	0.60	2.77	[135]
(MA)_3_(Sb_1−x_Sn_x_)_2_I	1.55	0.56	8.32	0.58	2.69	[136]
Cs_3_Sb_2_I_9_	2.05	0.72	5.21	0.39	1.49	[137]
(MA)_3_Sb_2_I_9_	2.14	0.89	1.0	0.55	0.5	[138]
Rb_3_Sb_2_I_9_	2.24	0.55	2.12	0.66	0.66	[139]
Rb_3_Sb_2_BR_9−x_Ix (P_x−0.9_)	2.02	0.55	4.25	0.595	1.37	[140]
(NH_4_)_3_Sb_2_I_9_	2.27	1.03	1.15	0.43	0.57	[141]
(MA)_3_Sb_2_I_9_	1.95	0.64	3.81	0.455	1.11	[142]
(MA)_3_Sb_2_I_9_:HI	1.95	0.62	5.41	0.68	2.04	[142]
Cs_3_Sb_2_I_9_	2.0	0.62	2.34	O.462	0.67	[142]
Cs_3_Sb_2_I_9_:HI	2.0	0.60	2.91	0.48	0.84	[142]
(MA)_3_Sb_2_I_9−x_Clx	2.11	0.53	4.43	0.58	1.37	[143]
(MA)_3_Sb_2_I_9−x_Clx:LITFSI	2.05	0.7	7.38	0.65	3.34	[143]
Cs_3_Bi_2_I_9_	2.2	0.85	2.15	0.60	1.09	[143]
(MA)_3_Bi_2_I_9_	2.1	0.68	0.52	0.33	0.12	[143]
(MA)_3_Bi_2_I_9−x_Cl_x_	2.4	0.04	0.18	0.38	0.003	[143]
(MA)_3_Bi_2_I_9_	-	0.83	3.00	0.79	1.64	[144]
(FA)_3_Bi_2_I_9_	2.19	0.48	0.11	0.46	0.022	[145]

## Abbreviations

0-D	zero-dimensional
1-D	one-dimensional
2-D	two-dimensional
3-D	three-dimensional
Eg	Bandgap
FA	Formamidinium
FF	fill factor
FTO	fluorine-doped tin oxide
ITO	indium tin oxide
Jsc	Short-circuit current
(LiTFSI)	bis(trifluoromethane)sulfonimide lithium
MA	methylammonium
PC71BM	[6,6]-phenyl C71butyric acid methyl-ester
PCBM	[6,6]-phenyl-C61-butyric acid methyl ester
PC61BM	[6,6]-phenyl-C61-butyric acid methyl ester
PCE	power conversion efficiency
PEDOT: PSS	poly(3,4-ethylenedioxythiophene) poly(styrenesulfonate)
PSCs	perovskite solar cells
PL	Photoluminescence
PV	Photovoltaics
SEM	Scanning electron microscope
spiro-OMeTAD	2,20,7,70-tetrakis-(*N*,*N*-di-*p*-methoxyphenylamine)9,90-spirobifluorene
TGA	Thermogravimetric analysis
Voc	open circuit voltage
XRD	X-ray diffraction
UV-vis	ultraviolet-visible absorption
